# Antimicrobial Resistance, β-Lactamases, and Virulence-Associated Genes in Clinical *Escherichia coli* Isolates from a Pediatric Tertiary-Care Hospital in Honduras

**DOI:** 10.3390/microorganisms14102257

**Published:** 2026-10-04

**Authors:** Nancy Alvarez-Corrales, Daniela Chavez-Bulnes, Bryan Ortiz, Jorge Carrasco, Juan Rosales-Tamashiro, Milena Videa Galo, Marco Moncada, María Félix Rivera, Daniela Rivera-Pineda, Lelany Pineda-García, Manuel G. Ballesteros-Monrreal

**Affiliations:** 1 Grupo de Investigación Científica en Microbiología One Health, Departamento de Microbiología, Escuela de Microbiología, Facultad de Ciencias, Universidad Nacional Autónoma de Honduras, Tegucigalpa 11101, Honduras; nancy.alvarez@unah.edu.hn; 2 Departamento de Biología Celular y Patología, Instituto de Neurociencias de Castilla y León (INCyL), Universidad de Salamanca, 37007 Salamanca, Spain; d.chavez@usal.es; 3 Instituto de Investigaciones en Microbiología, Facultad de Ciencias, Universidad Nacional Autónoma de Honduras, Tegucigalpa 11101, Honduras; bryan.ortiz@unah.edu.hn; 4 Departamento de Bioanálisis e Inmunología, Escuela de Microbiología, Facultad de Ciencias, Universidad Nacional Autónoma de Honduras, Tegucigalpa 11101, Honduras; jorge.carrasco@unah.edu.hn; 5 Departamento de Microbiología, Escuela de Microbiología, Facultad de Ciencias, Universidad Nacional Autónoma de Honduras, Tegucigalpa 11101, Honduras; jjrosalest@unah.edu.hn (J.R.-T.); milena.videa@unah.edu.hn (M.V.G.); 6 Laboratorio de Bacteriología, Hospital Escuela Universitario, Tegucigalpa 11101, Honduras; dr.marco_moncada@yahoo.es; 7 Departamento de Ciencias Fisiológicas, Facultad de Ciencias Médicas, Universidad Nacional Autónoma de Honduras, Tegucigalpa 11101, Honduras; maria.rivera@unah.edu.hn; 8 Facultad de Medicina, Universidad Católica de Honduras, Tegucigalpa 11101, Honduras; danielariverapineda@gmail.com; 9 Departamento de Ciencias Químico-Biológicas y Agropecuarias, Universidad de Sonora, Caborca 83600, Mexico

**Keywords:** *Escherichia coli*, urinary isolates, ExPEC, Honduras, virulence factors, hospital, pediatric patients

## Abstract

The co-occurrence of antimicrobial resistance and virulence in *Escherichia coli* threatens the treatment of pediatric infections, particularly in regions with limited molecular data. We characterized 67 clinical isolates recovered at a tertiary-care pediatric hospital in Tegucigalpa, Honduras, during 2017–2018. Antimicrobial susceptibility was assessed by disk diffusion; extended-spectrum β-lactamase (ESBL) and carbapenemase phenotypes by Etest; and β-lactam resistance and virulence-associated genes by PCR. Overall, 35.8% of isolates were multidrug-resistant. The highest resistance frequencies were observed for trimethoprim/sulfamethoxazole (65.7%), ciprofloxacin (37.3%), cefepime and ceftriaxone (35.8% each), and ceftazidime (34.3%). Of the 24 isolates resistant to third- and/or fourth-generation cephalosporins, 19 had a confirmed ESBL phenotype. One isolate exhibited a metallo-β-lactamase phenotype. A total of 23 isolates underwent molecular analysis, and at least one β-lactamase gene was detected in nine isolates; *bla*_TEM_ was detected in seven isolates, *bla*_SHV_ in two, and *bla*_NDM_ in one. In addition, 65 isolates (97.0%) carried virulence-associated genes, most commonly *fimH* (85.1%), *feoB* (74.6%), and *iucD* (67.2%). The marked co-occurrence observed among resistance phenotypes and among virulence-associated genes underscores the need to strengthen epidemiological and molecular surveillance of *E. coli* in the Honduran pediatric population.

## 1. Introduction

*Escherichia coli* (*E. coli*) is one of the leading bacterial pathogens associated with human infection that can cause conditions ranging from localized disease to invasive infection. In pediatric populations, it is most associated with urinary tract infections, bacteremia, sepsis, and meningitis, the severity of which may vary according to the patient’s clinical condition and the characteristics of the causative isolate [1,2,3,4]. The clinical diversity of these infections reflects the ability of *E. coli* to adapt to different host niches and to employ multiple mechanisms of colonization, persistence, and tissue damage. This versatility is supported by the extensive genetic diversity of the species and by the acquisition or loss of mobile genetic elements and virulence determinants, which can enable specific lineages to cause intestinal or extraintestinal disease.

In hospital settings, *E. coli* is among the microorganisms most frequently recovered from clinical specimens and is associated with urinary tract infections, intra-abdominal infections, bacteremia, sepsis, surgical-site infections, and other healthcare-associated infections [5,6]. The coexistence of virulence factors and antimicrobial resistance poses both epidemiological and therapeutic challenges because it may enhance bacterial persistence, restrict treatment options, and contribute to the circulation of difficult-to-control strains or high-risk lineages [6,7]. The combined distribution of these traits may vary across regions, population groups, phylogroups, and bacterial lineages; therefore, their characterization should be interpreted within the local epidemiological context.

The pathogenicity of *E. coli* depends on combinations of factors involved in adhesion, motility, toxin production, iron acquisition, biofilm formation, and immune evasion, and the repertoire of these determinants may vary among isolates and according to the source of infection [8,9]. These factors include adhesins encoded by genes such as *fimH*, *papC*, and *sfaD*/*focC*; toxins such as HlyA and Cnf-1; and iron-acquisition systems such as aerobactin and yersiniabactin, all of which contribute to bacterial colonization, persistence, and survival within the host [6,10,11,12,13,14,15]. Some of these genes may cluster within pathogenicity islands (PAIs), horizontally acquired genomic regions that carry combinations of virulence determinants. The co-occurrence of PAI-associated genes has also been documented in clinical *E. coli* isolates and may contribute to the development of complex virulence repertoires [16,17,18,19,20].

In addition to its virulence repertoire, *E. coli* is an important reservoir of antimicrobial resistance among Gram-negative bacteria and one of the major pathogens contributing to the global burden of antimicrobial resistance [21]. It also has a strong capacity to acquire and disseminate genes through plasmids, integrons, and transposons [21,22,23]. These genetic elements may simultaneously carry resistance determinants against multiple antimicrobial classes and, in some cases, virulence-associated genes, thereby promoting horizontal transfer and co-selection [24,25,26,27].

Among the resistance mechanisms of greatest clinical relevance is the production of extended-spectrum β-lactamases (ESBLs), particularly those belonging to the CTX-M, TEM, and SHV families, which compromise the activity of extended-spectrum cephalosporins and monobactams [28,29,30]. The spread of ESBL-producing isolates has increased the use of carbapenems, whereas the emergence of carbapenemases such as KPC, NDM, and OXA-48 has further reduced available therapeutic options [31,32]. The coexistence of these resistance mechanisms with broad virulence repertoires may promote bacterial persistence, the dissemination of successful lineages, and greater difficulty in controlling infections.

High frequencies of ESBL-producing *Enterobacterales* have been documented in Latin America, particularly in hospital settings, although their distribution varies across countries, healthcare facilities, and study populations [33]. In Honduras, ESBL-producing *E. coli* and multidrug-resistant *Enterobacterales* with resistance profiles affecting β-lactams, fluoroquinolones, and aminoglycosides have also been reported [33,34,35]. However, information integrating antimicrobial susceptibility, β-lactamase production, and the distribution of virulence-associated genes in clinical *E. coli* isolates from Honduran pediatric patients remains limited. A combined assessment of these features is needed to identify local resistance and virulence patterns and to strengthen microbiological surveillance.

To address this knowledge gap, this study aimed to characterize antimicrobial susceptibility, β-lactamase and carbapenemase phenotypes, and the distribution of 17 virulence-associated genes among clinical *E. coli* isolates recovered from pediatric patients treated at a tertiary-care hospital in Tegucigalpa, Honduras. These findings provide baseline information on antimicrobial resistance and virulence potential in *E. coli* circulating within the Honduran pediatric population.

## 2. Materials and Methods

### 2.1. Study Design and Ethical Considerations

An observational, descriptive, cross-sectional study was conducted using clinical isolates presumptively identified as *Escherichia coli*. The isolates were recovered during routine microbiological diagnosis of pediatric patients treated at Hospital Escuela Universitario, a tertiary-care hospital in Tegucigalpa, Honduras, between September 2017 and June 2018.

All available isolates that met the established microbiological criteria for identification and viability were included, regardless of specimen type or antimicrobial susceptibility profile. Only one isolate per patient was analyzed. Sociodemographic and hospital data, including age, gender, place of origin, and admission ward or service, were obtained from institutional clinical records and linked to the isolates through identification codes.

The study was conducted in accordance with the ethical principles of the Declaration of Helsinki. The protocol was approved by the Bioethics Committee of the Honduran Social Security Institute (IHSS) under registration number 006-CB-HE. Clinical information was handled confidentially and used exclusively for the purposes established in the research protocol.

### 2.2. Recovery, Phenotypic Identification, and Preservation of Isolates

Isolates presumptively identified as *Escherichia coli* were transferred to the Tropical Diseases Research Laboratory (LIET) for recovery and characterization. Upon arrival, each isolate was subcultured on blood agar and MacConkey agar and incubated aerobically at 37 °C for 24 h.

Preliminary biochemical identification included assessment of colony morphology, glucose and lactose fermentation, gas and hydrogen sulfide production, motility, citrate utilization, and urease, phenylalanine deaminase, lysine decarboxylase, ornithine decarboxylase, and tryptophanase activities, with the latter determined by indole production. Phenotypic identification was confirmed using the API^®^ 20E miniaturized system (bioMérieux, Marcy-l’Étoile, France) according to the manufacturer’s instructions.

### 2.3. Genomic DNA Extraction

Genomic DNA was extracted using the Wizard^®^ Genomic DNA Purification Kit (Promega, Madison, WI, USA) according to the manufacturer’s instructions. The extracted DNA was resuspended in 100 µL of rehydration solution and stored at −20 °C until use.

### 2.4. Molecular Confirmation of Escherichia coli

Species identification was confirmed by conventional PCR targeting *ybbW*, a gene encoding a receptor involved in allantoin metabolism that has been used as a species-specific marker for *E. coli*, according to previously reported protocols [36,37].

PCR was performed in a final volume of 25 µL containing 12.5 µL of 2 × PCR Master Mix (GoTaq® Green Master Mix Promega, Madison, WI, USA), 1 µL of each primer at 10 µM, 1.5 µL of genomic DNA, and nuclease-free water to complete the final volume. The primers *ybbW*-F (5′-TGATTGGCAAAATCTGGCCG-3′) and *ybbW*-R (5′-ATACTGGCAATCAGTACGCC-3′), which amplify a 667-bp fragment, were used.

The amplification conditions consisted of an initial denaturation at 95 °C for 4 min, followed by 37 cycles of 95 °C for 70 s, 60 °C for 70 s, and 72 °C for 1 min, with a final extension at 72 °C for 10 min. *Escherichia coli* ATCC 25922 was used as the positive control, and a reaction without template DNA was included as the negative control.

All PCR products generated in this study were separated on 2% agarose gels, stained with ethidium bromide at a final concentration of 0.5 µg/mL, and visualized using a Gel Doc™ EZ Imaging System (Bio-Rad Laboratories, Hercules, CA, USA).

### 2.5. Antimicrobial Susceptibility Testing

Antimicrobial susceptibility was determined by the Kirby–Bauer disk diffusion method. Inoculum preparation, plate inoculation, incubation, and measurement of inhibition-zone diameters were performed according to Clinical and Laboratory Standards Institute guidelines. Susceptibility categories (susceptible, intermediate, resistant) were assigned using the breakpoints established in CLSI M100, 36th edition (2026) [38], applied retrospectively to the original inhibition-zone diameters recorded for isolates collected in 2017–2018.

Eleven antimicrobial agents from the following categories were evaluated: penicillin/β-lactamase inhibitor combinations, amoxicillin/clavulanic acid (AMC, 20/10 µg); third-generation cephalosporins, ceftazidime (CAZ, 30 µg) and ceftriaxone (CRO, 30 µg); fourth-generation cephalosporins, cefepime (FEP, 30 µg); cephamycins, cefoxitin (CX, 30 µg); carbapenems, imipenem (IMI, 10 µg); fluoroquinolones, ciprofloxacin (CIP, 5 µg) and levofloxacin (LVX, 5 µg); aminoglycosides, amikacin (AMK, 30 µg); folate pathway inhibitors, trimethoprim/sulfamethoxazole (SXT, 1.25/23.75 µg); and nitrofurans, nitrofurantoin (NF, 300 µg for urine specimens only). Oxoid™ antimicrobial susceptibility testing discs (Thermo Fisher Scientific, Basingstoke, UK) were used. *Escherichia coli* ATCC 25922 was included as the quality-control strain.

Isolates were classified as non-multidrug-resistant (NMDR) or multidrug-resistant (MDR) according to the criteria proposed by Magiorakos. When an isolate was resistant to all antimicrobial agents and categories included in the available panel, but not all categories required to establish an XDR classification had been tested, it was classified as possibly extensively drug-resistant (pXDR) [39].

Isolates yielding an intermediate (I) category result by disk diffusion (observed for amikacin, *n* = 2, and amoxicillin/clavulanic acid, *n* = 6) were grouped with resistant (R) isolates as non-susceptible for the purposes of resistance frequency reporting, the resistance index (RI), and MDR/XDR classification, consistent with the criteria of Magiorakos et al.

### 2.6. Phenotypic Confirmation of Extended-Spectrum β-Lactamase Production

The extended-spectrum β-lactamase (ESBL)-producing phenotype was evaluated only in isolates resistant to third- and/or fourth-generation cephalosporins, according to CLSI criteria [38]. Phenotypic confirmation was performed using Etest^®^ strips (bioMérieux, Marcy L’Etoile, France) containing cefotaxime and cefotaxime/clavulanic acid gradients (CT/CTL; 0.25–16/0.016–1 µg/mL), which simultaneously determine the minimum inhibitory concentrations (MICs) of both agents.

The test was interpreted as positive when the ratio of the cefotaxime MIC to the cefotaxime/clavulanic acid MIC (CT MIC/CTL MIC) was ≥8. The presence of a phantom zone or deformation of the inhibition ellipse associated with clavulanic acid synergy was also considered indicative of ESBL production. Results were classified as indeterminate when one or both MIC values exceeded the measurement range of the strip and the CT/CTL ratio could not be calculated.

Cefoxitin resistance was considered a presumptive phenotypic indicator of AmpC β-lactamase production but was not interpreted as confirmation of this mechanism.

### 2.7. Phenotypic Detection of Metallo-β-Lactamases

The metallo-β-lactamase (MBL)-producing phenotype was evaluated in isolates resistant to imipenem according to CLSI criteria [38]. Detection was performed using Etest^®^ strips containing imipenem and imipenem/EDTA gradients (IP/IPI; 4–256/1–64 µg/mL) (bioMérieux, Marcy L’Etoile, France), which simultaneously determine the MIC of imipenem alone (IP; 4–256 µg/mL) and in combination with EDTA (IPI; 1–64 µg/mL).

The test was interpreted according to the manufacturer’s criteria by considering the ratio of the imipenem MIC to the imipenem/EDTA MIC (IP MIC/IPI MIC). The presence of a phantom zone or deformation of the inhibition ellipse associated with the effect of EDTA was also considered indicative of MBL production.

As a complementary assessment of carbapenem susceptibility in MBL-positive isolates, the ertapenem MIC was determined using Etest^®^ strips (bioMérieux, Marcy L’Etoile, France) with a measurement range of 0.002–32 µg/mL. Results were interpreted according to CLSI breakpoints [38].

### 2.8. Molecular Detection of β-Lactamase Genes

Molecular analysis included isolates with confirmed β-Lactamase production, isolates with indeterminate CT/CTL results in which ESBL production could not be excluded, and the metallo-β-lactamase-producing isolate. Isolates in which ESBL production phenotype was ruled out and that were classified exclusively as possible AmpC producers were excluded.

Molecular detection was performed by endpoint PCR according to previously published protocols [40,41,42]. The β-lactamase gene families *bla*_CTX-M_ Group IV according to Pitout [40], *bla*_TEM_ and *bla*_SHV_ and the carbapenemase genes *bla*_KPC_ and *bla*_NDM_ were investigated.

Each reaction was prepared in a final volume of 40 µL containing 20 µL of Green GoTaq^®^ Master Mix 2X (Promega, Madison, WI, USA), 4 µL of each forward and reverse primer prepared at a concentration of 0.2 µM, and 4 µL of template DNA. Primer sequences, annealing temperatures, specific amplification conditions, and expected amplicon sizes are provided in Table S1. PCR amplicons were not sequenced; therefore, detection of β-lactamase genes was interpreted at the gene-family level and did not allow identification of specific allelic variants.

### 2.9. Virulence-Associated Genes

All 67 *Escherichia coli* isolates were screened for 17 virulence-associated genes by endpoint PCR. The evaluated genes included *fimH*, *sfaD*/*focC*, *papG-II*, *papC*, *agn43*, *iha*, *fliCD*, *hlyA*, *sat*, *vat*, *cnf-1*, *iucD*, *fyuA*, *iutA*, *feoB*, *kpsM* and *traT*. These genes represent virulence-associated functions related to adhesion, motility, toxin production, iron acquisition, biofilm formation, and immune evasion. Detection was performed according to previously described protocols [16,43]. Primer sequences, gene distribution across multiplex reactions, expected amplicon sizes, and specific amplification conditions are provided in Table S1.

DNA from *Escherichia coli* CFT073, ATCC 25922, and the clinical isolate UPEC 39, which had been previously characterized for the genes under investigation [16], was used as positive-control material. A reaction without template DNA was included in each assay as a negative control.

### 2.10. Virulence and Resistance Index

A virulence index (VI) was calculated for each isolate to enable comparisons among the study groups. The VI was defined as the proportion of detected virulence-associated genes relative to the 17 genes included in the panel:

$${\mathrm{VI}}_{i}=\frac{V_{i}}{17}$$

where V_i_ is the number of virulence-associated genes detected in isolate i. The VI reflects overall virulence gene burden rather than equal contribution of each gene to host damage severity, consistent with the cumulative gene-count principle used in virulence scoring in ExPEC molecular epidemiology [44].

Similarly, a resistance index (RI) was calculated as the proportion of antimicrobial agents to which an isolate was resistant relative to the total number of agents actually tested:

$$RI_{i}=\frac{R_{i}}{A_{i}}$$

where R_i_ is the number of antimicrobial agents to which isolate i was resistant and A_i_ is the number of antimicrobial agents tested against isolate i.

Nitrofurantoin was included only in the calculation for isolates recovered from urine. For comparisons between urinary isolates and isolates from other clinical specimens, the RI was calculated using the ten antimicrobial agents tested against all isolates.

### 2.11. Statistical Analysis

Categorical variables were expressed as absolute frequencies and percentages, whereas continuous variables were summarized as mean ± standard deviation or median and interquartile range (Q1–Q3), depending on their distribution. Normality and homogeneity of variances for the virulence and resistance indices were assessed using the Shapiro–Wilk, Brown–Forsythe, and Bartlett tests. Comparisons by gender were performed using an independent-samples Student’s *t*-test or the Mann–Whitney U test, whereas comparisons across age groups and admission wards were conducted using one-way analysis of variance or the Kruskal–Wallis test, as appropriate. Isolates without age information were excluded from age-based analyses.

The frequencies of virulence-associated genes and antimicrobial resistance were compared using two-sided Fisher’s exact tests for gender and the Fisher–Freeman–Halton exact test for age groups and admission wards. Odds ratios with 95% confidence intervals were calculated for comparisons by gender, and *p*-values from multiple comparisons were adjusted using the Benjamini–Hochberg procedure. Nitrofurantoin was analyzed only among urinary isolates and was excluded from comparisons in which no variability was observed.

The association between the VI and RI was assessed using Spearman’s correlation. Co-occurrence between resistance phenotypes and among virulence-associated genes was analyzed using Pearson’s correlation coefficient for binary variables, equivalent to the phi coefficient, with 95% confidence intervals and two-sided *p*-values. Analyses were performed using GraphPad Prism 7.00, R version 4.5.0 through RStudio 2026.07.0+139, and JASP 0.19.3.0. Figures were generated in R using the ggplot2 (version 4.0.3), dplyr (version 1.2.1), and patchwork (version 1.3.2) packages. Values of *p* or adjusted *p* < 0.05 were considered statistically significant.

Given the sample size and the unequal numbers of isolates across some age and hospital-ward categories, subgroup analyses were interpreted as exploratory, particularly when involving groups with small numbers of isolates.

## 3. Results

### 3.1. Patient Sociodemographic Characteristics and Clinical Isolates

A total of 82 presumptive *E. coli* isolates were received at the Tropical Diseases Research Laboratory (LIET) during the study period, corresponding to all isolates submitted for this purpose without additional selection by the investigators. Fifteen isolates (12 urine, 2 epithelial exudate, and 1 blood culture) did not amplify the species-specific *ybbW* marker and were excluded, yielding a final study population of 67 participants (Table S2), with a predominance of female children (51/67; 76.1%). The median age was 6.0 years (Q1–Q3: 1.3–15.8), with a mean of 7.9 ± 6.9 years and a range of 0.016–18.0 years. The most represented age groups were 1–4 and 15–18 years old, each comprising 20 participants (29.9%). Children younger than 1 year 12 months and those aged 5–9 years accounted for 10 participants (14.9%) each (Table 1).

Regarding geographic origin, 23 participants (34.3%) were from the department of Francisco Morazán, and 25 (37.3%) resided in urban areas. The hospitalization ward was documented in 33 cases (49.3%). The Pediatric Emergency Department accounted for the largest number of admissions (33/67; 49.3%), followed by Pediatric Observation Ward (8/67; 11.9%) and the Gynecology and Obstetrics Emergency Department (6/67; 9.0%). Department of origin, area of residence, and hospitalization status were not specified in 50.7%, 52.2%, and 50.7% of the records, respectively (Table 1).

Regarding specimen source, most isolates were recovered from urine cultures (76.1%), followed by epithelial exudates (10.4%). Respiratory specimens and genital secretions yielded three isolates each (4.5%), whereas two isolates were recovered from blood cultures (3.0%) and one from a stool culture (1.5%).

### 3.2. Virulence Factors

#### 3.2.1. Number of Detected Genes and Virulence Index

Most isolates carried at least one virulence-associated gene: 65 of 67 (97.0%) were positive for one or more genes included in the panel, whereas two isolates (3.0%) were negative for all genes evaluated. The number of detected genes per isolate ranged from 0 to 15, with a mean of 7.0 ± 3.3. The broadest virulence repertoire was observed in isolate 75-U679, which carried 15 of the 17 genes evaluated.

**Figure 1:**
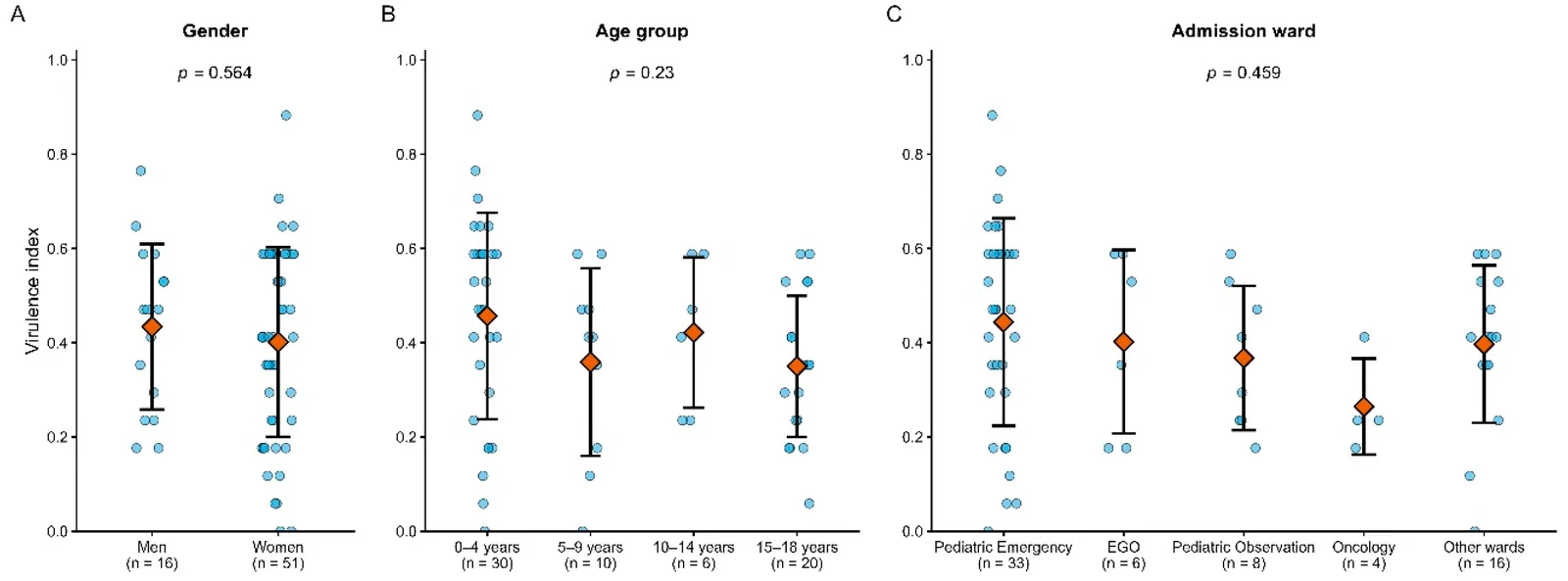
Virulence index of the clinical *Escherichia coli* isolates according to the characteristics of the study population. (**A**) Virulence index according to gender. (**B**) Virulence index according to age group. (**C**) Virulence index according to admission ward or service. Each blue point represents an individual isolate; the orange diamond indicates the mean virulence index of each group, and the error bars represent the standard deviation. The virulence index was calculated as the number of detected genes divided by the 17 genes evaluated. Differences were assessed using an unpaired Student’s *t*-test for gender and one-way analysis of variance for age group and admission ward or service.

Based on this variability, the virulence index (VI) was compared across groups defined by patient characteristics. Isolates from male patients had a slightly higher mean VI than those from female patients, with values of 0.434 ± 0.176 (*n* = 16) and 0.401 ± 0.201 (*n* = 51), respectively (Figure 1A). However, this difference was not statistically significant [*t*(65) = 0.579; *p* = 0.564].

The distribution of the VI was then examined across age groups. The isolate from the participant with missing age information was excluded from this analysis. The highest mean VI was observed in the 0–4-year group (0.457 ± 0.219; *n* = 30), followed by the 10–14-year group (0.422 ± 0.160; *n* = 6), the 5–9-year group (0.359 ± 0.199; *n* = 10), and the 15–18-year group (0.350 ± 0.149; *n* = 20) (Figure 1B). Despite these differences, no statistically significant variation was observed among age groups [*F*(3,62) = 1.475; *p* = 0.230].

Finally, the VI was compared according to admission ward or service. Mean values were 0.444 ± 0.220 for the Pediatric Emergency Department (*n* = 33), 0.402 ± 0.195 for the Gynecology and Obstetrics Emergency Department (EGO; *n* = 6), 0.397 ± 0.167 for other services (*n* = 16), 0.368 ± 0.153 for the Pediatric Observation Ward (*n* = 8), and 0.265 ± 0.102 for Oncology (*n* = 4) (Figure 1C). As in the previous comparisons, the observed differences were not statistically significant [*F*(4,62) = 0.919; *p* = 0.459].

#### 3.2.2. Overall Detection Frequency of Virulence-Associated Genes

**Figure 2:**
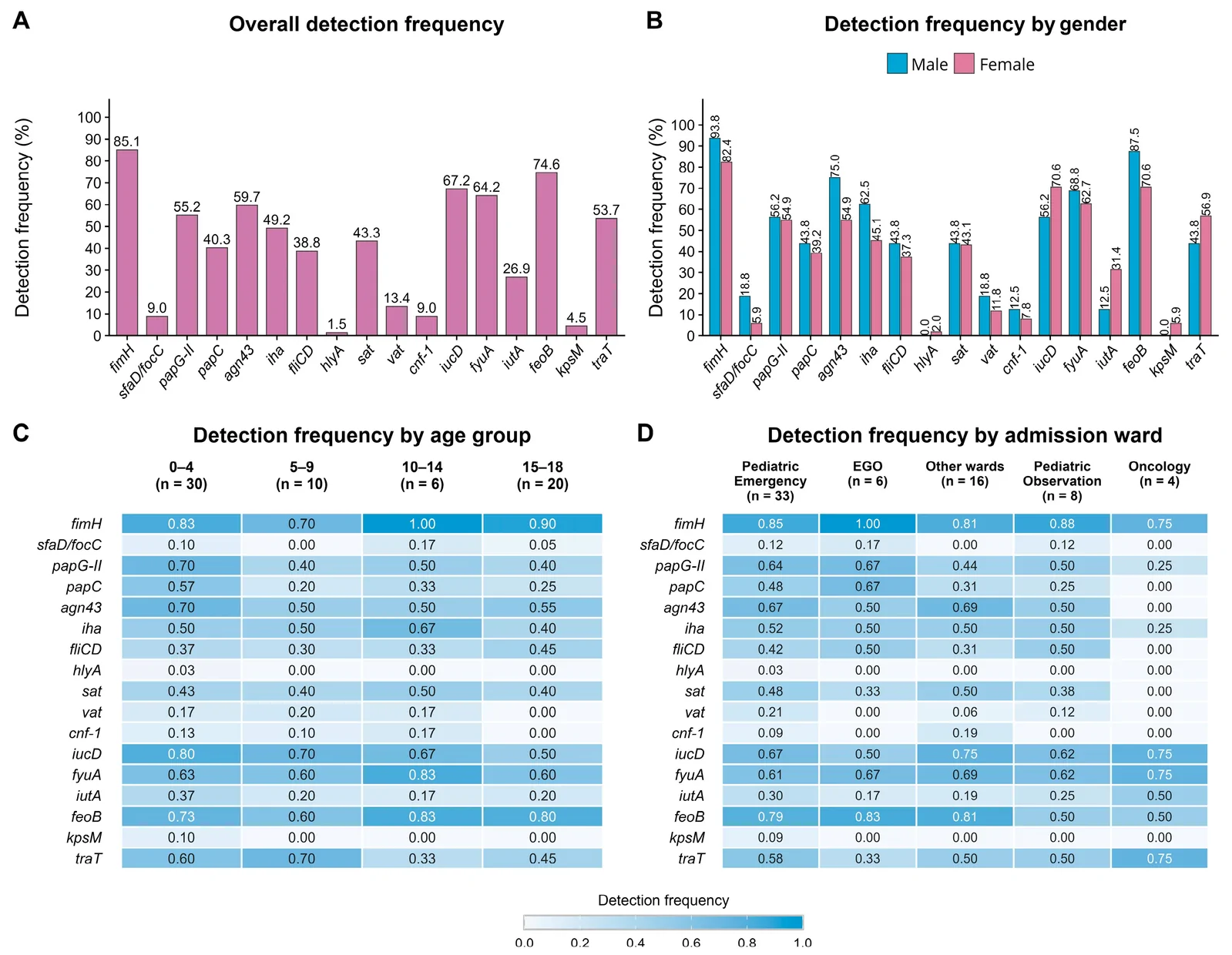
Detection frequency of virulence-associated genes among the clinical *Escherichia coli* isolates from pediatric patients. (**A**) Overall detection frequency of the 17 genes evaluated in the 67 isolates. (**B**) Detection frequency according to gender: male (*n* = 16) and female (*n* = 51). (**C**) Detection frequency according to age group: 0–4 years (*n* = 30), 5–9 years (*n* = 10), 10–14 years (*n* = 6), and 15–18 years (*n* = 20); one isolate was excluded because age information was unavailable. (**D**) Detection frequency according to admission ward or service: Pediatric Emergency Department (*n* = 33), Gynecology and Obstetrics Emergency Department (EGO; *n* = 6), other services (*n* = 16), Pediatric Observation Unit (*n* = 8), and Oncology (*n* = 4). In the heat maps, greater color intensity indicates a higher proportion of positive isolates. Percentages were calculated using the number of isolates included in each group as the denominator. Comparisons were performed using Fisher’s exact test for gender and the Fisher–Freeman–Halton exact test for age group and admission ward or service. The Benjamini–Hochberg procedure was applied to adjust for multiple comparisons.

Because the virulence index did not differ significantly by gender, age group, or admission ward, the individual distribution of the 17 virulence-associated genes was examined. Among adhesion-related genes, *fimH* showed the highest detection frequency (85.1%). The motility-associated gene *fliCD* was detected in 38.8% of the isolates, whereas *sat* was the most frequently detected toxin-associated gene (43.3%). Among iron-acquisition genes, *feoB* predominated (74.6%), while *traT* showed the highest frequency among genes associated with immune evasion (53.7%) (Figure 2A).

#### 3.2.3. Detection Frequency by Gender, Age Group, and Admission Ward

When isolates were stratified by gender, *fimH* was the most frequently detected gene in isolates from both male and female patients, with frequencies of 93.8% and 82.4%, respectively. Among isolates from male patients, *feoB* (87.5%) and *agn43* (75.0%) were also frequent. In isolates from female patients, the highest frequencies after *fimH* were observed for *iucD* and *feoB* (70.6% each), followed by *fyuA* (62.7%). None of the 17 genes was significantly associated with gender after adjustment for multiple comparisons. The lowest *p*-value was observed for *sfaD*/*focC* (*p* = 0.142; adjusted *p* = 0.825) (Figure 2B).

The distribution of virulence-associated genes was also examined across the 0–4-year (*n* = 30), 5–9-year (*n* = 10), 10–14-year (*n* = 6), and 15–18-year (*n* = 20) age groups. *fimH* was the most frequently detected gene in all groups, with frequencies of 83.3%, 70.0%, 100.0%, and 90.0%, respectively. In the 0–4-year group, *iucD* (80.0%) and *feoB* (73.3%) were also frequent; in the 5–9-year group, *iucD* and *traT* were detected in 70.0% of isolates each; in the 10–14-year group, *fyuA* and *feoB* were detected in 83.3% each; and in the 15–18-year group, *feoB* and *fyuA* were detected in 80.0% and 60.0%, respectively. Despite these differences in frequency, no significant associations were detected between age group and detection of any of the 17 genes after adjustment for multiple comparisons. The lowest *p*-value was observed for *papC* (*p* = 0.070; adjusted *p* = 0.650) (Figure 2C).

Gene distribution also varied across admission wards, although several genes remained among the most frequently detected in multiple services. In the Pediatric Emergency Department, the most frequent genes were *fimH* (84.8%), *feoB* (78.8%), and *iucD* and *agn43* (66.7% each). In the Gynecology and Obstetrics Emergency Department (EGO), *fimH* (100.0%), *feoB* (83.3%), and *papG-II*, *papC*, and *fyuA* (66.7% each) predominated. In other services, *fimH* and *feoB* showed the highest frequencies (81.3% each), followed by *iucD* (75.0%). In Pediatric Observation, *fimH* was detected in 87.5% of isolates, while *iucD* and *fyuA* were each detected in 62.5%. In Oncology, *fimH*, *iucD*, *fyuA*, and *traT* were each detected in 75.0% of isolates. No significant associations were detected between admission ward and detection frequency of any gene after adjustment for multiple comparisons. The lowest *p*-value was observed for *agn43* (*p* = 0.107; adjusted *p* = 1.000) (Figure 2D).

#### 3.2.4. Virulence Profiles of Clinical Isolates

The combination of the 17 genes evaluated yielded 58 distinct virulence profiles among the 65 isolates in which at least one virulence-associated gene was detected, indicating substantial heterogeneity in gene repertoire composition. Most profiles were identified in a single isolate, whereas five were detected in two or more isolates.

Among the recurring profiles, some were found exclusively in isolates from the same admission ward, particularly in the Pediatric Emergency Department, whereas others were distributed across different hospital services. The composition and distribution of the five recurring profiles are shown in Table 2. Shared profiles were interpreted descriptively and were not considered evidence of clonal or epidemiological relatedness among isolates.

#### 3.2.5. Co-Occurrence of Virulence-Associated Genes

The tendency of virulence-associated genes to occur together among the clinical *Escherichia coli* isolates was examined. Using Pearson’s correlation coefficient for binary variables, equivalent to the phi coefficient, positive associations were identified among genes involved in adhesion, toxin production, iron acquisition, and immune evasion.

The strongest correlation was observed between *papG-II* and *papC* (*r* = 0.740; 95% CI: 0.608–0.832; *p* < 0.001), both of which are components of the P-fimbrial system. A strong positive correlation was also identified between *sfaD*/*focC* and *vat* (*r* = 0.643; 95% CI: 0.476–0.765; *p* < 0.001).

Positive associations were also observed among *sat*, *agn43*, and *iha*. Detection of *sat* correlated with *iha* (*r* = 0.646; 95% CI: 0.480–0.767; *p* < 0.001) and *agn43* (*r* = 0.533; 95% CI: 0.336–0.686; *p* < 0.001), while *agn43* also correlated with *iha* (*r* = 0.627; 95% CI: 0.455–0.754; *p* < 0.001).

Genes involved in iron acquisition also showed positive co-occurrence. *iucD* correlated with *fyuA* (*r* = 0.472; 95% CI: 0.261–0.640; *p* < 0.001), *iha* (*r* = 0.435; 95% CI: 0.217–0.611; *p* < 0.001), and *iutA* (*r* = 0.424; 95% CI: 0.204–0.603; *p* < 0.001). Positive correlations were also observed between *iucD* and *traT* (*r* = 0.435; 95% CI: 0.217–0.611; *p* < 0.001), and between *fyuA* and *iha* (*r* = 0.425; 95% CI: 0.205–0.603; *p* < 0.001).

Overall, these findings indicate that several virulence-associated genes tended to co-occur within the isolates analyzed. The complete correlation matrix for all 17 virulence-associated genes is provided in Figure S1.

### 3.3. Antibiotic Resistance

The number of antimicrobial agents to which each isolate was resistant ranged from 0 to 10, with a mean of 2.72 ± 2.68. The mode was resistance to one antimicrobial agent, observed in 19 isolates (28.4%). Isolate 7-E2608 showed the broadest resistance profile and was resistant to all 10 agents included in the panel evaluated for non-urinary isolates. In contrast, 16 isolates (23.9%) were susceptible to all antimicrobial agents tested. The association between the resistance index (RI) and virulence index (VI) was also assessed using Spearman’s correlation coefficient; however, no statistically significant correlation was observed (*r_s_* = 0.30; 95% CI: −0.054–0.594; *p* = 0.08).

According to the criteria proposed by Magiorakos [39], 24 of the 67 isolates (35.8%) were classified as multidrug-resistant (MDR), whereas the remaining 43 (64.2%) were classified as non-multidrug-resistant (NMDR). MDR/NMDR status showed an overall association with admission ward according to the two-sided Fisher–Freeman–Halton exact test (*p* = 0.043). However, none of the pairwise comparisons between wards remained significant after Benjamini–Hochberg adjustment (*p* > 0.05); therefore, no specific between-ward differences were identified.

**Figure 3:**
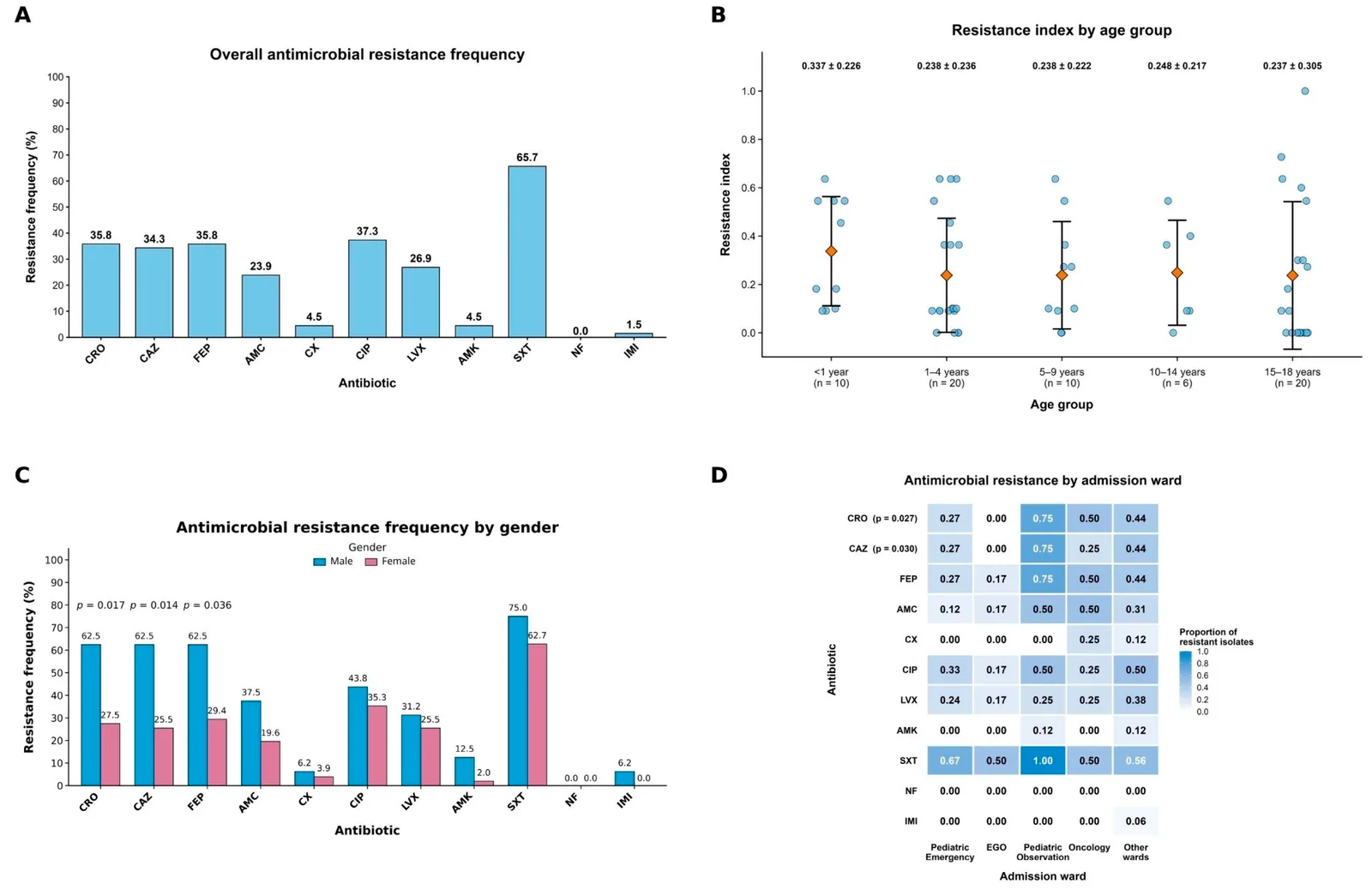
Antimicrobial resistance patterns among clinical *Escherichia coli* isolates from pediatric patients. (**A**) Overall resistance frequency. (**B**) Resistance index by age group. Boxes show the median and interquartile range, whiskers extend to values within 1.5 × IQR, and points represent individual isolates. The isolate with missing age information was excluded. Differences were evaluated using the Kruskal–Wallis test. (**C**) Resistance frequency by gender; displayed values are Benjamini–Hochberg-adjusted *p*-values from significant two-sided Fisher’s exact tests. (**D**) Proportion of resistant isolates by admission ward. Cell values range from 0 to 1, with darker color indicating higher resistance frequency. Displayed values for CRO and CAZ are adjusted *p*-values from the global Fisher–Freeman–Halton test. Nitrofurantoin was excluded from inferential analyses because no resistant isolates were detected. CRO, ceftriaxone; CAZ, ceftazidime; FEP, cefepime; AMC, amoxicillin/clavulanic acid; CX, cefoxitin; CIP, ciprofloxacin; LVX, levofloxacin; AMK, amikacin; SXT, trimethoprim/sulfamethoxazole; NF, nitrofurantoin; IMI, imipenem; EGO, Gynecology and Obstetrics Emergency Department.

Among the 67 isolates, the highest resistance frequency was observed for SXT (44/67; 65.7%), followed by CIP (25/67; 37.3%), FEP and CRO (24/67; 35.8% each), and CAZ (23/67; 34.3%) (Figure 3A). Resistance to AMC and LVX was detected in 23.9% and 26.9% of isolates, respectively. The lowest frequencies were observed for CX and AMK (4.5% each) and IMI (1.5%). Nitrofurantoin was evaluated only in the 51 urinary isolates, and none showed resistance.

Because RI values were not normally distributed across all groups, comparisons by age group and admission ward were performed using the Kruskal–Wallis test, whereas the comparison by gender was performed using the Mann–Whitney U test. Accordingly, RI values are reported as medians and interquartile ranges (Q1–Q3).

The median RI was 0.318 (Q1–Q3: 0.098–0.546; *n* = 10) among children younger than 1 year, 0.100 (Q1–Q3: 0.091–0.432; *n* = 20) in the 1–4-year group, 0.186 (Q1–Q3: 0.068–0.409; *n* = 10) in the 5–9-year group, 0.227 (Q1–Q3: 0.068–0.436; *n* = 6) in the 10–14-year group, and 0.091 (Q1–Q3: 0.000–0.484; *n* = 20) in the 15–18-year group. No statistically significant differences were observed among age groups [Kruskal–Wallis, *H*(4) = 3.122; *p* = 0.5376].

By gender, isolates from male patients had a median RI of 0.382 (Q1–Q3: 0.093–0.546; *n* = 16), whereas those from female patients had a median RI of 0.100 (Q1–Q3: 0.000–0.364; *n* = 51). The RI was significantly higher among isolates from male patients (Mann–Whitney U = 265; two-sided *p* = 0.0328).

Resistance to ceftriaxone, ceftazidime, and cefepime was also significantly more frequent among isolates from male than female patients (Figure 3C). For ceftriaxone, the corresponding frequencies were 62.5% and 27.5% (adjusted *p* = 0.017), and isolates from male patients had approximately 4.3-fold higher odds of resistance (OR = 4.30; 95% CI: 1.16–17.39). Similarly, ceftazidime resistance was detected in 62.5% of isolates from male patients and 25.5% of those from female patients (adjusted *p* = 0.014; OR = 4.74; 95% CI: 1.27–19.37). For cefepime, the corresponding frequencies were 62.5% and 29.4% (adjusted *p* = 0.036; OR = 3.91; 95% CI: 1.07–15.70). The confidence intervals were wide, largely because of the limited number of isolates from male patients (*n* = 16). Therefore, although the ORs indicated associations of considerable magnitude, the estimates should be interpreted cautiously.

When RI was compared across admission wards, the highest median was observed among isolates from Pediatric Observation [0.500 (Q1–Q3: 0.236–0.546); *n* = 8], followed by other services [0.227 (Q1–Q3: 0.025–0.627); *n* = 16], Oncology [0.182 (Q1–Q3: 0.000–0.636); *n* = 4], Pediatric Emergency [0.091 (Q1–Q3: 0.045–0.364); *n* = 33], and the Gynecology and Obstetrics Emergency Department (EGO) [0.091 (Q1–Q3: 0.000–0.211); *n* = 6]. No statistically significant differences were identified among wards [Kruskal–Wallis, *H*(4) = 8.284; *p* = 0.0817].

Nevertheless, resistance frequencies differed significantly among admission wards for ceftriaxone (adjusted *p* = 0.027) and ceftazidime (adjusted *p* = 0.030) (Figure 3D). For both agents, the highest frequency was observed among isolates from Pediatric Observation (75.0%), followed by other services (43.8%) and Pediatric Emergency (27.3%); no resistant isolates were detected in EGO. In pairwise comparisons, resistance in Pediatric Observation was significantly more frequent than in Pediatric Emergency (adjusted *p* = 0.035) and EGO (adjusted *p* = 0.010) for both antimicrobial agents. No significant between-ward differences were found for the remaining agents evaluated (adjusted *p* > 0.05). Nitrofurantoin was excluded from inferential analyses because no resistant isolates were detected.

In addition to the differences observed in resistance frequency, a strong co-occurrence was identified among resistance phenotypes to third- and fourth-generation cephalosporins. Ceftriaxone resistance was closely associated with resistance to ceftazidime (*r* = 0.968; 95% CI: 0.948–0.980) and cefepime (*r* = 0.968; 95% CI: 0.949–0.980). A similarly strong association was observed between ceftazidime and cefepime (*r* = 0.937; 95% CI: 0.899–0.961; *p* < 0.001 for all comparisons). Overall, 24 isolates were resistant to at least one of the third- or fourth-generation cephalosporins evaluated and were subsequently included in the phenotypic characterization of β-lactamase production.

Cephalosporin resistance was also associated with fluoroquinolone resistance. Ciprofloxacin resistance showed positive associations with resistance to ceftriaxone (*r* = 0.647; 95% CI: 0.481–0.768), ceftazidime (*r* = 0.677; 95% CI: 0.522–0.789), and cefepime (*r* = 0.617; 95% CI: 0.443–0.747; *p* < 0.001 for all three comparisons). Similarly, levofloxacin resistance was positively associated with resistance to ceftriaxone (*r* = 0.390; 95% CI: 0.165–0.576; *p* = 0.001), ceftazidime (*r* = 0.413; 95% CI: 0.192–0.594; *p* < 0.001), and cefepime (*r* = 0.368; 95% CI: 0.140–0.559; *p* = 0.002). These findings indicate that resistance to cephalosporins and fluoroquinolones frequently co-occurred within the same isolates. The complete correlation matrix for all antimicrobial agents evaluated is provided in Figure S1.

#### 3.3.1. Phenotypic Characterization of β-Lactam Resistance Mechanisms

Among the 67 *Escherichia coli* isolates, 24 (35.8%) were resistant to at least one of the cephalosporins evaluated. Of these, 22 exhibited an ESBL-associated screening phenotype: 21 were classified as presumptive ESBL producers and one as a combined ESBL/possible AmpC producer. Of the two remaining isolates, one was classified as a possible AmpC producer and one as a possible carbapenemase producer.

#### 3.3.2. ESBL- and Possible AmpC-Producing Phenotypes

The 22 isolates with a presumptive ESBL or ESBL/possible AmpC phenotype were recovered mainly from the Pediatric Emergency Department (9/33; 27.3%) and Pediatric Observation (6/8; 75.0%). Although the Pediatric Emergency Department contributed the largest absolute number of isolates, the Pediatric Observation Ward showed the highest proportion of isolates with these phenotypes. The remaining seven isolates were distributed across Labor and Delivery, Oncology, Autopsy, NCP, Pediatric Medicine, Renal Therapy, and the Septic Unit, with one isolate recovered from each service.

All 22 isolates were evaluated using the CT/CTL Etest^®^, based on comparison of the minimum inhibitory concentrations of cefotaxime and cefotaxime/clavulanic acid. ESBL production was confirmed in 19 isolates, corresponding to 86.4% of these 22 isolates and 28.4% of the 67 isolates included in the study. Among the 19 confirmed isolates, 13 exhibited a phantom zone or deformation of the inhibition ellipse indicative of synergy with clavulanic acid.

Six isolates with a presumptive ESBL phenotype were identified in Pediatric Observation. Five were confirmed by CT/CTL Etest^®^, whereas isolate 21-U3640 yielded an indeterminate result. Thus, confirmed ESBL producers accounted for 62.5% (5/8) of the isolates recovered from this ward. In Pediatric Emergency, eight ESBL-producing isolates were confirmed, representing 24.2% (8/33) of the isolates recovered from that service.

Of the three isolates with a presumptive ESBL or ESBL/possible AmpC phenotype that were not confirmed, two yielded indeterminate results. Isolate 21-U3640 was recovered from a 3-year-old female patient admitted to Pediatric Observation, whereas isolate 124-U1492 was recovered from a 5-month-old male patient treated in the Pediatric Emergency Department. In both cases, the MICs of cefotaxime and cefotaxime/clavulanic acid exceeded the measurement ranges of the strips; therefore, the CT/CTL ratio could not be calculated, and ESBL production could neither be confirmed nor excluded.

Isolate 112-U1239, recovered from a 4-year-old female patient admitted to Renal Therapy and initially classified as having a combined ESBL/possible AmpC phenotype, tested negative for ESBL production by CT/CTL Etest^®^. The cefotaxime MIC was 0.25 µg/mL, the cefotaxime/clavulanic acid MIC was 0.23 µg/mL, and the CT/CTL ratio was 1.08. This isolate was also resistant to cefoxitin, consistent with its presumptive AmpC phenotype.

In addition, isolate 41-U93, recovered from a 15-year-old female patient admitted to Oncology and classified as a possible AmpC producer, was evaluated by CT/CTL Etest^®^ and yielded an indeterminate result. This isolate was also resistant to cefoxitin. Because the CT/CTL Etest^®^ assesses the inhibitory effect of clavulanic acid on ESBLs, the indeterminate result did not allow ESBL production to be confirmed or excluded in this isolate. Although cefoxitin resistance in isolates 112-U1239 and 41-U93 was compatible with an AmpC phenotype, this mechanism was not confirmed by a specific test.

All four isolates with negative or indeterminate CT/CTL results were recovered from urine cultures and had resistance indices ranging from 0.545 to 0.727. Isolates 21-U3640 and 124-U1492 were resistant to seven and six of the 11 antimicrobial agents included in the index calculation, with RI values of 0.636 and 0.545, respectively. Isolate 112-U1239 was resistant to seven agents (RI = 0.636), whereas isolate 41-U93 was resistant to eight (RI = 0.727).

All four isolates were resistant to ceftriaxone, ceftazidime, cefepime, ciprofloxacin, and trimethoprim/sulfamethoxazole. Overall, although ESBL production could not be confirmed in three of these isolates and was ruled out in one, all exhibited concurrent resistance to multiple antimicrobial agents.

For subsequent molecular analysis, the 19 confirmed ESBL producers and the three isolates with indeterminate CT/CTL results (21-U3640, 41-U93, and 124-U1492) were retained, whereas isolate 112-U1239 was excluded because ESBL production was ruled out by CT/CTL Etest^®^.

#### 3.3.3. Metallo-β-Lactamase-Producing Phenotype

Only one isolate (1/67; 1.5%), identified as 7-E2608, exhibited a presumptive carbapenemase-producing phenotype. This isolate was recovered from an exudate obtained from a 17-year-old male patient admitted to Pediatric Orthopedics.

By IP/IPI Etest^®^, the isolate showed an imipenem MIC of 4 µg/mL and an imipenem/EDTA MIC of 1 µg/mL, corresponding to an IP/IPI ratio of 4. Although this ratio was below the positivity threshold of ≥8, deformation of the inhibition ellipse indicative of synergy with EDTA was observed; therefore, the isolate was interpreted as a metallo-β-lactamase producer. The isolate also showed an ertapenem MIC of 4 µg/mL, corresponding to the resistant category.

Isolate 7-E2608 exhibited the broadest resistance profile in the collection: it was resistant to all ten antimicrobial agents evaluated for non-urinary isolates and had a resistance index of 1.000. Although the metallo-β-lactamase phenotype, ertapenem resistance, and complete resistance to the panel evaluated were consistent with an extensively drug-resistant profile, the panel did not include representatives of all antimicrobial categories required by Magiorakos et al. to establish a definitive XDR classification (e.g., polymyxins, tigecycline, and fosfomycin were not evaluated). The isolate was therefore conservatively classified as possibly extensively drug-resistant (pXDR).

#### 3.3.4. Molecular Detection of β-Lactamase Genotypes

A total of 23 isolates underwent molecular analysis, comprising the 19 confirmed ESBL producers, the three isolates with indeterminate CT/CTL results in which ESBL production could not be excluded, and the single metallo-β-lactamase-producing isolate. At least one of the evaluated β-lactamase genes was detected in nine of these 23 isolates (39.1%). Among these nine molecularly positive isolates, *bla*_TEM_ was detected in seven (77.8%), *bla*_SHV_ in two (22.2%), and *bla*_NDM_ in one (11.1%). One isolate carried both *bla*_TEM_ and *bla*_SHV_ (Table 3).

The *bla*_NDM_-positive isolate was 7-E2608, recovered from an exudate from a 17-year-old male patient treated in Pediatric Orthopedics. This isolate was resistant to all ten antimicrobial agents evaluated and showed an imipenem MIC of 4 µg/mL and an imipenem/EDTA MIC of 1 µg/mL, together with deformation of the inhibition ellipse consistent with metallo-β-lactamase production. It was also resistant to ertapenem, with an MIC of 4 µg/mL. Detection of *bla*_NDM_ was concordant with the observed resistance phenotype. The *bla*_KPC_ gene was not detected in any of the isolates tested.

## 4. Discussion

*Escherichia coli* is one of the bacterial pathogens of greatest clinical relevance in pediatric populations worldwide. According to the most recent analysis of the global burden of disease covering 1990–2021, with projections to 2050, *E. coli* ranks among the leading pathogens associated with sepsis-related deaths in children aged 0–14 years, together with *Streptococcus pneumoniae*, *Klebsiella pneumoniae*, *Staphylococcus aureus*, and *Pseudomonas aeruginosa*. It has also been identified as a high-burden pathogen for which no licensed vaccine is currently available [45]. This epidemiological relevance, together with its role as a major etiological agent of urinary tract infections, bacteremia, and other extraintestinal infections in children, underscores the need for local characterization to support clinical management and epidemiological surveillance in pediatric populations such as that of Honduras.

Although clinical source alone is insufficient to establish an ExPEC or UPEC pathotype, interpretation of this collection was not based solely on specimen origin. The isolates were recovered during routine diagnostic evaluation of pediatric patients; hospitalization was documented in 33 cases, and molecular characterization included 17 virulence-associated genes widely described in extraintestinal and uropathogenic *E. coli*. Therefore, considering the predominance of urinary isolates, the hospital-based pediatric context, and the virulence repertoire investigated, the findings can reasonably be discussed within the broader biological framework of extraintestinal pathogenic *E. coli* (ExPEC), which includes uropathogenic *E. coli* (UPEC) and strains associated with other extraintestinal infections. This context is relevant because, to our knowledge, no previous Honduran study has focused exclusively on the molecular characterization of *E. coli* in a pediatric population while integrating virulence, resistance phenotypes, and resistance genotypes. The closest available report from Honduras involved a cohort of uropathogenic isolates recovered from patients of different ages [43].

The Honduran cohort described by Ortiz et al. [43] provides a direct comparison between pediatric and adult populations and supports the interpretation of the present findings in the context of other Latin American pediatric populations. In that study, isolates from boys aged 6–12 years carried a significantly greater burden of virulence genes than isolates from adolescents and adults. A pattern of high virulence in pediatric populations was also documented in a cohort of 178 pediatric urinary isolates from Mexico City, in which phylogroup B2, commonly associated with a greater virulence burden, predominated and 60.7% of isolates exhibited an MDR profile [46]. Taken together, the direction of the descriptive trend observed in the present collection toward a higher virulence index in the 0–4-year age group is consistent with patterns reported in other pediatric populations. However, this difference was not statistically significant and should not be interpreted as evidence of an age-associated effect in the present collection.

In addition to the descriptive variation observed across age groups, virulence-gene frequencies also varied across admission wards or services. Isolates from the Gynecology and Obstetrics Emergency Department exhibited high frequencies of *papG-II*, *papC*, and *fyuA* (66.7% each). Given the established role of *papG-II* in renal adhesion and colonization, its high frequency in this subgroup is biologically consistent with traits associated with adaptation to the urinary tract. However, these differences were not statistically significant, and the small size of this group (*n* = 6) precludes a definitive association.

The marked heterogeneity of virulence profiles observed in this study, 58 distinct profiles among 65 positive isolates, and the significant correlations between functionally related genes, particularly *papG-II*/*papC* within the P-fimbrial system and *sfaD*/*focC*/*vat*, are consistent with findings previously reported by the same research group in Mexican uropathogenic isolates. Ballesteros-Monrreal et al. (2021) [16] identified similar statistically significant co-occurrences among virulence genes and proposed that they might reflect the presence of pathogenicity islands (PAIs), including those described in the reference UPEC strains CFT073, J96, and 536. Subsequently, Ballesteros-Monrreal et al. (2024) [17] confirmed the presence of these PAIs in related isolates by PCR targeting specific PAI markers, strengthening the hypothesis proposed in the earlier study.

The present study did not include amplification of PAI-specific markers and therefore cannot directly confirm their presence in this collection. Nevertheless, the observed patterns of gene co-occurrence, particularly *papG-II*/*papC* and *sfaD*/*focC*/*vat*, are consistent with the genomic organization previously confirmed by the same research group. These co-occurrence patterns are compatible with, but do not demonstrate, similar genetic arrangements in the current collection and therefore support targeted investigation of these elements in future Honduran isolates. Functional convergence of this type has also been demonstrated in ColV-type plasmids carried by ExPEC, in which virulence and antimicrobial resistance genes may reside on the same mobile genetic element, facilitating their co-dissemination under selective pressure [47].

Regarding antimicrobial resistance, Ortiz et al. (2025) [43] found that isolates from pediatric boys had a substantially lower resistance burden than those from adult men, whereas girls younger than 12 years showed particularly high prevalences of resistance to trimethoprim/sulfamethoxazole, amoxicillin/clavulanic acid, and ampicillin, which are commonly used first-line agents in empirical pediatric treatment. This latter pattern is especially relevant to the present study, in which SXT showed the highest resistance frequency (65.7%). This finding suggests that high resistance to first-line antimicrobial agents in pediatric populations may represent a recurring regional phenomenon.

Notably, none of the 51 urinary isolates was resistant to nitrofurantoin. This finding is consistent with a 10-year surveillance study of pediatric cohorts in the United States, in which nitrofurantoin maintained comparatively low resistance rates relative to other commonly used agents, including SXT [48]. It is also consistent with a recent global meta-analysis of nitrofurantoin resistance in UPEC, which reported substantially lower resistance prevalences than those observed for other first-line agents [49]. In the local context, the absence of nitrofurantoin resistance may also be related, at least in part, to its limited use for pediatric urinary tract infections at Hospital Escuela, which may result in lower bacterial exposure to this antimicrobial [50]. In contrast, SXT, which is commonly prescribed in primary care settings, showed the highest resistance frequency in the present study. Taken together, these findings suggest that, despite the declining activity of several traditionally recommended first-line antibiotics, nitrofurantoin retained high in vitro activity against urinary isolates in this collection. This observation is also consistent with the more recent findings reported by Ortiz et al. (2025) [43] in Honduras, supporting the persistence of this susceptibility pattern over time. Nevertheless, its current suitability for empirical treatment of uncomplicated pediatric urinary tract infections should be evaluated using contemporary local surveillance data.

Unlike virulence, antimicrobial resistance showed a statistically significant association with patient gender. Isolates from male patients had a significantly higher resistance index than those from female patients, as well as significantly higher frequencies of resistance to ceftriaxone, ceftazidime, and cefepime. This association is consistent with the findings of Ajabnoor et al. (2025) [51], who reported in a seven-year retrospective analysis from Saudi Arabia that male gender was significantly associated with a higher frequency of resistance among urinary *E. coli* isolates, regardless of age group.

The proportion of isolates resistant to third- and fourth-generation cephalosporins (35.8%) and the strong co-occurrence of resistance to these agents and fluoroquinolones are consistent with the regional epidemiology reported for Central America. In a review of hospital-based surveillance in Latin America, Guzmán-Blanco et al [52] identified Honduras, together with Guatemala and Mexico, as countries with particularly high rates of third-generation cephalosporin resistance in *E. coli*. Thus, the present findings are consistent with a regional pattern documented more than a decade ago. Similarly, a genomic surveillance study conducted at a pediatric hospital in Lima, Peru, identified a considerable proportion of MDR isolates in both clinical and environmental samples [53], indicating that this problem is not restricted to the Honduran setting. A broader genomic survey of uropathogenic *E. coli* across six Latin American countries similarly documented substantial genomic diversity among circulating resistance and virulence determinants [54], underscoring the heterogeneity of *E. coli* populations responsible for extraintestinal infections across the region.

The overall association between MDR/NMDR classification and admission ward, although not maintained in pairwise comparisons after adjustment for multiple testing, also coincided descriptively with the highest median resistance index and the highest frequencies of ceftriaxone and ceftazidime resistance among isolates from Pediatric Observation. However, the small number of isolates recovered from this ward limits the interpretation of this pattern.

Regarding the frequency of resistance genes, only 39.1% of isolates with a presumptive ESBL or positive metallo-β-lactamase phenotype carried at least one of the β-lactamase genes detected in this study. The phenotype–genotype discordance observed in the remaining isolates may reflect the presence of additional β-lactamase determinants not included in the molecular panel. Although *bla*_CTX-M_ was investigated, the primer pair used specifically targeted CTX-M Group IV according to the classification of Pitout et al., which includes CTX-M-9, -13, -14, -16 to -19, -21, and -27. Therefore, the absence of amplification does not exclude the presence of *bla*_CTX-M_ genes belonging to other CTX-M groups not targeted by this assay. Future studies in this setting could usefully combine molecular screening for *bla*_CTX-M-15_ with sequence type (ST) determination of circulating isolates, given the well-documented global association between this variant and the high-risk clone ST131 [7,23]. Such an approach could help clarify whether the phenotype–genotype discordance observed in the present collection is attributable to the circulation of *bla*_CTX-M-15_ carrying lineages not captured by the molecular panel used here, a question of particular epidemiological relevance in a country such as Honduras, where molecular data on *E. coli* in pediatric infections remain scarce. Similarly, although *bla*_TEM_ and *bla*_SHV_ were detected in isolates exhibiting resistance to extended-spectrum cephalosporins, including isolates with confirmed ESBL production, endpoint PCR identifies these gene families but does not discriminate between narrow-spectrum and ESBL-associated allelic variants. Therefore, the detection of *bla*_TEM_ or *bla*_SHV_ is compatible with the observed β-lactam resistance phenotype, but it cannot establish that the detected allele itself was responsible for ESBL production. Amplicon sequencing would be required to determine the specific TEM or SHV variant. Other β-lactamase genes or resistance mechanisms may also have contributed to the observed cephalosporin-resistance phenotypes.

The identification of isolate 7-E2608, a metallo-β-lactamase producer carrying *bla*_NDM_ and resistant to all ten antimicrobial agents evaluated, as well as to ertapenem, represents a sentinel finding of particular clinical and epidemiological relevance. NDM-producing *E. coli* has been documented in pediatric hospital settings elsewhere, including cases of pediatric bloodstream infection in China [55] and the cross-border emergence of NDM-5 variants in the European Union [56]. These reports indicate that the spread of carbapenemase-producing *E. coli* in pediatric populations is an increasing global concern.

The significant co-occurrence of cephalosporin and fluoroquinolone resistance is also compatible with the possible circulation of multidrug-resistance plasmids carrying both *bla* genes and plasmid-mediated quinolone resistance determinants such as *qnr*. Such an association has been documented among foodborne *E. coli* isolates in Germany, where *qnr*-positive isolates were identified predominantly among ESBL producers [57].

Although the isolates in this collection were recovered in 2018, the findings reported here, including the early identification of an NDM-producing isolate and the high resistance to first-line antimicrobial agents in pediatric patients, anticipated patterns increasingly documented in subsequent regional and global studies. This supports the value of the present dataset as a historical baseline for surveillance of virulence and antimicrobial resistance in Honduras, particularly when interpreted alongside more recent international evidence.

This study has several limitations. The relatively small sample size, historical collection period, cross-sectional design, and single-center setting may limit the precision and generalizability of specific frequency estimates. In addition, because susceptibility categories were assigned using CLSI M100, 36th edition (2026) breakpoints applied retrospectively to inhibition-zone diameters recorded in 2017–2018, the resistance frequencies reported here may not be directly comparable to studies from the same period, or to regional studies such as Guzmán-Blanco et al. (2014) [52], that applied the breakpoints in effect at their respective times of data collection. Because this was a retrospective study based on previously collected clinical isolates and the clinical information available in the corresponding records, no direct patient contact occurred and additional clinical variables could not be prospectively obtained. Consequently, information such as prior antimicrobial exposure and complete clinical criteria to distinguish infection from colonization was not available for all cases; these constraints reflect limitations of data availability inherent to the retrospective design. Molecular limitations included the absence of amplicon sequencing for *bla*_TEM_ and *bla*_SHV_, which prevented identification of specific allelic variants; the absence of *qnr* detection in the molecular panel; and the lack of a specific confirmatory assay for AmpC production in isolates with compatible phenotypes. Accordingly, the *bla*_TEM_ and *bla*_SHV_ results should be interpreted as gene-family detection and not as identification of specific ESBL-associated variants. Similarly, the *bla*_CTX-M_ primer pair used in this study specifically targets the CTX-M-9 cluster [40] and does not detect the CTX-M-1 cluster, which includes *bla*_CTX-M-15_, the most prevalent ESBL determinant worldwide and the variant most closely associated with the pandemic ST131 lineage. Consequently, the absence of amplification observed here may underestimate the true prevalence of *bla*_CTX-M_ among the ESBL-positive isolates evaluated. The predominance of urinary isolates and the inclusion of different clinical specimen types reflect the composition of this hospital-based collection and should be considered when interpreting the findings. These limitations restrict the molecular resolution of some resistance mechanisms and the extrapolation of specific frequencies to other hospital settings in Honduras. Nevertheless, this study represents one of the first molecular characterizations of virulence and antimicrobial resistance in pediatric *Escherichia coli* isolates from Honduras. Together with the findings reported by Ortiz et al. (2025) [43] in a broader population, it provides relevant epidemiological evidence from a country in which molecular surveillance of *E. coli* remains limited and contributes to the still-scarce information on virulence and resistance in Central America compared with other regions of Latin America. The detection of an NDM-producing isolate in a pediatric patient further emphasizes the need for continued molecular surveillance of this pathogen at both national and regional levels.

## 5. Conclusions

Clinical *Escherichia coli* isolates recovered from pediatric patients at a tertiary-care hospital in Honduras exhibited substantial diversity in virulence determinants and antimicrobial resistance profiles. The predominance of genes associated with adhesion, iron acquisition, toxin production, and immune evasion highlights the pathogenic potential of these isolates. In addition, the co-occurrence of functionally related virulence genes may reflect their organization within shared genetic regions or pathogenicity islands (PAIs). Although their physical location was not determined in this study, these patterns support further investigation of the presence, composition, and diversity of PAIs among circulating isolates. Accordingly, statistical co-occurrence should not be interpreted as evidence of physical genetic linkage.

Resistance to trimethoprim/sulfamethoxazole, cephalosporins, and fluoroquinolones, together with the significant co-occurrence of resistance to different cephalosporin generations and ciprofloxacin, suggests the possible circulation of epidemiologically relevant multidrug-resistance plasmids. These elements may facilitate the co-dissemination of β-lactam and fluoroquinolone resistance determinants; however, confirmation will require conjugation assays, plasmid typing, and genomic sequencing. In this context, the detection of *bla*_NDM_ in a pediatric metallo-β-lactamase-producing pXDR isolate represents a particularly relevant finding because of its potential therapeutic and epidemiological implications.

In practical terms, the limited use of nitrofurantoin for pediatric urinary tract infections at Hospital Escuela reflects national AIEPI-based prescribing guidelines and institutional drug availability rather than an established clinical contraindication in pediatric patients. Given the complete absence of nitrofurantoin resistance among urinary isolates in this collection, its potential inclusion as an empirical option within pediatric urinary tract infection treatment schemes merits further exploration, contingent on confirmation with contemporary local surveillance data.

This study is among the first hospital-based investigations in Honduras focused exclusively on pediatric patients to integrate phenotypic and molecular characterization of antimicrobial resistance with the analysis of virulence factors in clinical *E. coli* isolates. The findings provide baseline information on circulating resistance and virulence determinants and underscore the need to strengthen microbiological surveillance, antimicrobial stewardship, and genomic characterization of priority isolates. Identification of plasmids, PAIs, and other mobile genetic elements will help determine whether the observed associations result from horizontal gene transfer, clonal expansion, or a combination of both processes.

## Figures and Tables

**Table 1:** Sociodemographic and hospital characteristics of the study population (N = 67).

Characteristic	Measure/Category	Overall (N = 67)
**Age in years**	Median (Q1–Q3)	6.0 (1.3–15.8)
	Mean ± SD	7.9 ± 6.9
	Range	0.016–18.0
**Age group,** ***n*** **(%)**	<1 year	10 (14.9)
	1–4 years	20 (29.9)
	5–9 years	10 (14.9)
	10–14 years	6 (9.0)
	15–18 years	20 (29.9)
	Not specified	1 (1.5)
**Gender,** ***n*** **(%)**	Female	51 (76.1)
	Male	16 (23.9)
**Department of origin,** ***n*** **(%)**	Francisco Morazán	23 (34.3)
	Other Departments	10 (14.9)
	Not specified	34 (50.7)
**Area of residence,** ***n*** **(%)**	Urban	25 (37.3)
	Rural	7 (10.4)
	Not specified	35 (52.2)
**Hospitalized patient,**	Yes	33 (49.3)
***n*** **(%)**	Not specified	34 (50.7)
**Admission ward or service,** ***n*** **(%)**	Emergency Department	33 (49.3)
	Observation Unit	8 (11.9)
	Gynecology and Obstetrics Emergency Department	6 (9.0)
	Oncology	4 (6.0)
	Labor and Delivery	2 (3.0)
	Orthopedics	2 (3.0)
	Renal Therapy	2 (3.0)
	Other services	10 (14.9)

Percentages were calculated using the total study population (N = 67) as the denominator. Age data were available for 66 participants. Q1–Q3: first and third quartiles; SD: standard deviation. The lower limit of the age range (0.016 years) corresponds to 6 days. “Other departments” included El Paraíso (*n* = 3), La Paz (*n* = 2), Intibucá, Lempira, Olancho, Valle, and Yoro (*n* = 1 each). “Other services” included Autopsy, Pediatric Surgery, Critical Care, Infant Care, Pediatric Medicine, NCP, Pediatric Nephrology, Nutrition, Septic Unit, and the Pediatric Intensive Care Unit (*n* = 1 each).

**Table 2:** Virulence profiles detected in two or more clinical *Escherichia coli* isolates and their distribution according to admission ward or service.

Virulence Profile	*n*	Admission Ward or Service
*papG-II*	3	Pediatric Emergency Department (*n* = 3)
*fimH*, *papG-II*, *papC*, *iucD*, *fyuA*, *sat*, *agn43*, *iha*, *iutA*, *feoB*	3	Pediatric Emergency Department (*n* = 2); Gynecology and Obstetrics Emergency Department (EGO; *n* = 1)
*fimH*, *papG-II*, *papC*, *iucD*, *sat*, *agn43*, *traT*, *iha*, *iutA*, *feoB*	2	Pediatric Emergency Department (*n* = 2)
*fimH*, *iucD*, *fyuA*, *fliCD*, *sat*, *feoB*	2	Renal Therapy (*n* = 1); Critical Care (*n* = 1)
*fimH*, *papG-II*, *papC*, *iucD*, *fyuA*, *sat*, *agn43*, *traT*, *iha*, *feoB*	2	Gynecology and Obstetrics Emergency Department (EGO; *n* = 1); Septic Unit (*n* = 1)

Only virulence profiles detected in two or more isolates are shown. *n*, number of isolates; *fimH*, type 1 fimbrial adhesin; *papG-II*, P fimbrial adhesin allele II; *papC*, P fimbrial assembly; *iucD*, aerobactin biosynthesis; *fyuA*, yersiniabactin receptor; *fliCD*, flagellin-associated genes; *sat*, secreted autotransporter toxin; *agn43*, antigen 43; *iha*, iron-regulated gene A homolog adhesin; *iutA*, ferric aerobactin receptor; *feoB*, ferrous iron transport protein B; *traT*, outer membrane protein associated with serum resistance. Identical virulence profiles were interpreted descriptively and do not necessarily indicate clonal or epidemiological relatedness among isolates.

**Table 3:** Clinical, phenotypic, and molecular characteristics of *Escherichia coli* isolates positive for at least one β-lactamase gene evaluated.

Isolate	Gender	Age (Years)	Admission Ward	Specimen	Phenotype	*bla*_TEM_	*bla*_SHV_	*bla*_NDM_	*bla*_KPC_	*bla*_CTX-M_
7-E2608	M	17	Pediatric Orthopedics	Exudate	pXDR/MBL	−	−	+	−	-
39-U85	F	14	Oncology	Urine	MDR/ESBL	+	+	−	−	-
61-U435	F	0.016	Pediatric Observation	Urine	MDR/ESBL	+	−	−	−	-
73-E349	M	17	Autopsy	Exudate	MDR/ESBL	−	+	−	−	-
79-U829	F	1.42	Pediatric Emergency	Urine	MDR/ESBL	+	−	−	−	-
90-U979	F	1.58	Pediatric Emergency	Urine	MDR/ESBL	+	−	−	−	-
98-U1359	M	2	Pediatric Emergency	Urine	MDR/ESBL	+	−	−	−	-
103-U1108	F	6	Pediatric Medicine	Urine	MDR/ESBL	+	−	−	−	-
116-G6713	F	16	Septic Unit	Genital secretion	MDR/ESBL	+	−	−	−	-

MDR: multidrug-resistant; pXDR: possibly extensively multidrug-resistant; ESBL: extended-spectrum β-lactamase; MBL: metallo-β-lactamase; +, gene detected; −, gene not detected.

## Data Availability

The original contributions presented in this study are included in the article/Supplementary Materials.

## Supplementary Materials

The following supporting information can be downloaded at: https://www.mdpi.com/article/10.3390/microorganisms14102257/s1. Table S1. Primers used in this study [16,36,37,40,41,42,58] a: *E. coli* CFT073; b: *E. coli* ATCC 25922; c: UPEC 39. Table S2. Complete Clinical, Phenotypic, and Molecular Dataset of the *Escherichia coli* Isolate Collection. Figure S1. Pairwise correlation matrices among antimicrobial resistance phenotypes and among virulence-associated genes. Figure S1A. Values shown are Pearson’s correlation coefficients for binary variables (equivalent to the phi coefficient), calculated as described in Section 2.10 of the main text. Color intensity is proportional to the strength of the correlation. Asterisks indicate statistical significance (*, *p* < 0.05; **, *p* < 0.01; ***, *p* < 0.001). These correlations are indicative of co-occurrence patterns rather than definitive evidence of physical genetic linkage or a shared underlying resistance or virulence mechanism. Antibiotic abbreviations: CRO, ceftriaxone; CAZ, ceftazidime; FEP, cefepime; AMC, amoxicillin/clavulanic acid; CX, cefoxitin; CIP, ciprofloxacin; LVX, levofloxacin; AMK, amikacin; SXT, trimethoprim/sulfamethoxazole; IMI, imipenem. Virulence gene abbreviations are as defined in the main text. Figure S1B. Pairwise correlation matrix of the 17 virulence-associated genes evaluated in this study. Values shown are Pearson’s correlation coefficients for binary variables (equivalent to the phi coefficient), calculated as described in Section 2.10 of the main text. Color intensity is proportional to the strength of the correlation. Asterisks indicate statistical significance (*, *p* < 0.05; **, *p* < 0.01; ***, *p* < 0.001). These correlations are indicative of co-occurrence patterns rather than definitive evidence of physical genetic linkage or a shared underlying virulence mechanism. Gene abbreviations: *fimH*, type 1 fimbrial adhesin; *sfaD/focC*, S/F1C fimbrial adhesin; *papG-II*, P fimbrial adhesin allele II; *papC*, P fimbrial assembly; *hlyA*, alpha-hemolysin; *kpsM*, group II capsule export; *iucD*, aerobactin biosynthesis; *fyuA*, yersiniabactin receptor; *fliCD*, flagellin-associated genes; sat, secreted autotransporter toxin; *agn43*, antigen 43; *vat*, vacuolating autotransporter toxin; *cnf-1*, cytotoxic necrotizing factor 1; *traT*, outer membrane protein associated with serum resistance; *iha*, iron-regulated gene A homolog adhesin; *iutA*, ferric aerobactin receptor; *feoB*, ferrous iron transport protein B.

## References

[1] Basmaci R., Bonacorsi S., Bidet P., Biran V., Aujard Y., Bingen E., Béchet S., Cohen R., Levy C. (2015). *Escherichia coli* Meningitis Features in 325 Children from 2001 to 2013 in France. Clin. Infect. Dis..

[2] Gibbs A.A., Laupland K.B., Edwards F., Ling W., Channon-Wells S., Harley D., Falster K., Paterson D.L., Harris P.N.A., Irwin A.D. (2024). Trends in Enterobacterales Bloodstream Infections in Children. Pediatrics.

[3] Malaure C., Geslain G., Birgy A., Bidet P., Poilane I., Allain M., Liberge M., Khattat N., Sikias P., Bonacorsi S. (2024). Early-Onset Infection Caused by *Escherichia coli* Sequence Type 1193 in Late Preterm and Full-Term Neonates. Emerg. Infect. Dis..

[4] Nhu N.T.K., Phan M.-D., Hancock S.J., Peters K.M., Alvarez-Fraga L., Forde B.M., Andersen S.B., Miliya T., Harris P.N.A., Beatson S.A. (2024). High-Risk *Escherichia coli* Clones That Cause Neonatal Meningitis and Association with Recrudescent Infection. Elife.

[5] Pitout J.D.D. (2012). Extraintestinal Pathogenic *Escherichia coli*: A Combination of Virulence with Antibiotic Resistance. Front. Microbiol..

[6] Sora V.M., Meroni G., Martino P.A., Soggiu A., Bonizzi L., Zecconi A. (2021). Extraintestinal Pathogenic *Escherichia coli*: Virulence Factors and Antibiotic Resistance. Pathogens.

[7] Nicolas-Chanoine M.-H., Bertrand X., Madec J.-Y. (2014). *Escherichia coli* ST131, an Intriguing Clonal Group. Clin. Microbiol. Rev..

[8] Manges A.R., Geum H.M., Guo A., Edens T.J., Fibke C.D., Pitout J.D.D. (2019). Global Extraintestinal Pathogenic *Escherichia coli* (ExPEC) Lineages. Clin. Microbiol. Rev..

[9] Lüthje P., Brauner A. (2014). Virulence Factors of Uropathogenic *E. coli* and Their Interaction with the Host. Adv. Microb. Physiol..

[10] Flores-Mireles A.L., Walker J.N., Caparon M., Hultgren S.J. (2015). Urinary Tract Infections: Epidemiology, Mechanisms of Infection and Treatment Options. Nat. Rev. Microbiol..

[11] Ambite I., Butler D., Wan M.L.Y., Rosenblad T., Tran T.H., Chao S.M., Svanborg C. (2021). Molecular Determinants of Disease Severity in Urinary Tract Infection. Nat. Rev. Urol..

[12] Ballesteros-Monrreal M.G., Mendez-Pfeiffer P., Barrios-Villa E., Arenas-Hernández M.M.P., Enciso-Martínez Y., Sepúlveda-Moreno C.O., Bolado-Martínez E., Valencia D. (2023). Uropathogenic *Escherichia coli* in Mexico, an Overview of Virulence and Resistance Determinants: Systematic Review and Meta-Analysis. Arch. Med. Res..

[13] Wang C., Li Q., Lv J., Sun X., Cao Y., Yu K., Miao C., Zhang Z.-S., Yao Z., Wang Q. (2020). Alpha-Hemolysin of Uropathogenic *Escherichia coli* Induces GM-CSF-Mediated Acute Kidney Injury. Mucosal Immunol..

[14] Sun X., Yang J., Deng X., Wei Y., Wang C., Guo Y., Yang H., Yang L., Miao C., Lv J. (2022). Interactions of Bacterial Toxin CNF1 and Host JAK1/2 Driven by Liquid-Liquid Phase Separation Enhance Macrophage Polarization. mBio.

[15] Galardini M., Clermont O., Baron A., Busby B., Dion S., Schubert S., Beltrao P., Denamur E. (2020). Major Role of Iron Uptake Systems in the Intrinsic Extra-Intestinal Virulence of the Genus Escherichia Revealed by a Genome-Wide Association Study. PLoS Genet..

[16] Ballesteros-Monrreal M.G., Arenas-Hernández M.M.P., Barrios-Villa E., Juarez J., Álvarez-Ainza M.L., Taboada P., De la Rosa-López R., Bolado-Martínez E., Valencia D. (2021). Bacterial Morphotypes as Important Trait for Uropathogenic *E. coli* Diagnostic; a Virulence-Phenotype-Phylogeny Study. Microorganisms.

[17] Ballesteros-Monrreal M.G., Mendez-Pfeiffer P., Ortíz B., Bolado-Martínez E., Álvarez-Ainza M.L., Enciso-Martínez Y., Arenas-Hernández M.M.P., Diaz-Murrieta B., Barrios-Villa E., Valencia D. (2024). Uropathogenic *E. coli* and Hybrid Pathotypes in Mexican Women with Urinary Tract Infections: A Comprehensive Molecular and Phenotypic Overview. Curr. Issues Mol. Biol..

[18] Royer G., Clermont O., Marin J., Condamine B., Dion S., Blanquart F. (2023). Epistatic Interactions between the High Pathogenicity Island and Other Iron Uptake Systems Shape *Escherichia coli* Extra-Intestinal Virulence. Nat. Commun..

[19] Desvaux M., Dalmasso G., Beyrouthy R., Barnich N., Delmas J., Bonnet R. (2020). Pathogenicity Factors of Genomic Islands in Intestinal and Extraintestinal *Escherichia coli*. Front. Microbiol..

[20] Biggel M., Xavier B.B., Johnson J.R., Nielsen K.L., Frimodt-Møller N., Matheeussen V., Goossens H., Moons P., Van Puyvelde S. (2020). Horizontally Acquired PapGII-Containing Pathogenicity Islands Underlie the Emergence of Invasive Uropathogenic *Escherichia coli* Lineages. Nat. Commun..

[21] Naghavi M., Vollset S.E., Ikuta K.S., Swetschinski L.R., Gray A.P., Wool E.E., Aguilar G.R., Mestrovic T., Smith G., Han C. (2024). Global Burden of Bacterial Antimicrobial Resistance 1990–2021: A Systematic Analysis with Forecasts to 2050. Lancet.

[22] World Health Organization (2024). WHO Bacterial Priority Pathogens List, 2024: Bacterial Pathogens of Public Health Importance to Guide Research, Development and Strategies to Prevent and Control Antimicrobial Resistance.

[23] Pitout J.D.D., DeVinney R. (2017). *Escherichia coli* ST131: A Multidrug-Resistant Clone Primed for Global Domination. F1000Res.

[24] da Silva G.J., Mendonça N. (2012). Association between Antimicrobial Resistance and Virulence in *Escherichia coli*. Virulence.

[25] Calhau V., Domingues S., Ribeiro G., Mendonça N., Da Silva G.J. (2015). Interplay between Pathogenicity Island Carriage, Resistance Profile and Plasmid Acquisition in Uropathogenic *Escherichia coli*. J. Med. Microbiol..

[26] Juraschek K., Malekzadah J., Malorny B., Käsbohrer A., Schwarz S., Meemken D., Hammerl J.A. (2022). Characterization of QnrB-Carrying Plasmids from ESBL- and Non-ESBL-Producing *Escherichia coli*. BMC Genom..

[27] Wang Y., Dagan T. (2024). The Evolution of Antibiotic Resistance Islands Occurs within the Framework of Plasmid Lineages. Nat. Commun..

[28] Cantón R., González-Alba J.M., Galán J.C. (2012). CTX-M Enzymes: Origin and Diffusion. Front. Microbiol..

[29] Masoud S.M., Abd El-Baky R.M., Aly S.A., Ibrahem R.A. (2021). Co-Existence of Certain ESBLs, MBLs and Plasmid Mediated Quinolone Resistance Genes among MDR *E. coli* Isolated from Different Clinical Specimens in Egypt. Antibiotics.

[30] Husna A., Rahman M.M., Badruzzaman A., Sikder M.H., Islam M.R., Rahman M.T., Alam J., Ashour H.M. (2023). Extended-Spectrum Beta-Lactamases (ESBL): Challenges and Opportunities. Biomedicines.

[31] Logan L.K., Weinstein R.A. (2017). The Epidemiology of Carbapenem-Resistant *Enterobacteriaceae*: The Impact and Evolution of a Global Menace. J. Infect. Dis..

[32] Boyd S.E., Holmes A., Peck R., Livermore D.M., Hope W. (2022). OXA-48-like Beta-Lactamases: Global Epidemiology, Treatment Options, and Development Pipeline. Antimicrob. Agents Chemother..

[33] Gales A.C., Castanheira M., Jones R.N., Sader H.S. (2012). Antimicrobial Resistance among Gram-Negative Bacilli Isolated from Latin America: Results from SENTRY Antimicrobial Surveillance Program (Latin America, 2008–2010). Diagn. Microbiol. Infect. Dis..

[34] Padgett D., Luque M.T., Rivera D.M., Galindo C., Zepeda L., Hernández A. (2011). Resistencia Antimicrobiana En Bacterias Aisladas En El Instituto Hondureño de Seguridad Social. Rev. Med. Hondur..

[35] Pineda-García L., Tzoc E., Rivera M.F., Herrera L., Moncada M. (2017). Caracterización Clínica y Epidemiológica En Pacientes Con Infección Por *Enterobacteriaceae* Productoras de Beta-Lactamasas de Espectro Extendido (BLEE), Hospital Escuela Universitario, Tegucigalpa, Honduras, Año 2013. Rev. Cienc. Y Tecnol..

[36] Carreón E. (2019). Estudio Molecular de La Resistencia y Virulencia de Cepas de *Escherichia coli* Productoras de β-Lactamasas de Espectro Extendido Aisladas de Vegetales Crudos. Master’s Thesis.

[37] Walker D.I., McQuillan J., Taiwo M., Parks R., Stenton C.A., Morgan H., Mowlem M.C., Lees D.N. (2017). A Highly Specific *Escherichia coli* QPCR and Its Comparison with Existing Methods for Environmental Waters. Water Res..

[38] Clinical and Laboratory Standards Institute (CLSI) (2026). Performance Standards for Antimicrobial Susceptibility Testing. CLSI Supplement M100.

[39] Magiorakos A.P., Srinivasan A., Carey R.B., Carmeli Y., Falagas M.E., Giske C.G., Harbarth S., Hindler J.F., Kahlmeter G., Olsson-Liljequist B. (2012). Multidrug-Resistant, Extensively Drug-Resistant and Pandrug-Resistant Bacteria: An International Expert Proposal for Interim Standard Definitions for Acquired Resistance. Microbiology.

[40] Pitout J.D.D., Hossain A., Hanson N.D. (2004). Phenotypic and Molecular Detection of CTX-M-β-Lactamases Produced by *Escherichia coli* and *Klebsiella* spp. J. Clin. Microbiol..

[41] Dallenne C., Da Costa A., Decré D., Favier C., Arlet G. (2010). Development of a Set of Multiplex PCR Assays for the Detection of Genes Encoding Important β-Lactamases in *Enterobacteriaceae*. J. Antimicrob. Chemother..

[42] Hou X., Song X., Ma X., Zhang S., Zhang J. (2015). Molecular Characterization of Multidrug-Resistant *Klebsiella pneumoniae* Isolates. Braz. J. Microbiol..

[43] Ortiz B., Ballesteros-Monrreal M.G., Galindo C., Rivera D., Rivera L., Pérez F., Maldonado V., Valle E., Estrada A., Mendez-Pfeiffer P. (2025). Comprehensive Molecular and Phenotypic Profiling of Uropathogenic *Escherichia coli* in a Honduran Healthcare Setting: Virulence, Resistance and Phylogeny. Front. Microbiol..

[44] Yang W.-T., Chiu I.-J., Huang Y.-T., Liu P.-Y. (2022). Comparative Genomics Revealed Fluoroquinolone Resistance Determinants and OmpF Deletion in Carbapenem-Resistant *Escherichia coli*. Front. Microbiol..

[45] Zhu W., Jiang P., Yu J., Shen T., Yang X., Xu Y., Jin S., Jiang Y. (2026). Childhood Sepsis Burden: Pathogens/Antimicrobial-Resistant Bacteria, 1990–2021 and 2050 Forecasts. Front. Cell. Infect. Microbiol..

[46] Luna-Pineda V.M., Ochoa S.A., Cruz-Córdova A., Cázares-Domínguez V., Reyes-Grajeda J.P., Flores-Oropeza M.A., Arellano-Galindo J., Hernández-Castro R., Flores-Encarnación M., Ramírez-Vargas A. (2018). Features of Urinary *Escherichia coli* Isolated from Children with Complicated and Uncomplicated Urinary Tract Infections in Mexico. PLoS ONE.

[47] Lian Z.J., Nhu N.T.K., Ravi C., Chang C., Martinez-Roman I., Phan M.-D., Schembri M.A. (2025). Convergence of Plasmid-Driven Virulence and Antibiotic Resistance in *Escherichia coli*. Nat. Commun..

[48] Mahajan S., Kanwar N., Morgan G.M., Mendes R.E., Lee B.R., Banerjee D., Selvarangan R. (2024). Antimicrobial Susceptibility Trends in *E. coli* Causing Pediatric Urinary Tract Infections in the United States. Pathogens.

[49] Larkin C., Valappil S.P., Palanisamy N. (2025). Global Prevalence of Nitrofurantoin-Resistant Uropathogenic *Escherichia coli* (UPEC) in Humans: A Systematic Review and Meta-Analysis. J. Antimicrob. Chemother..

[50] Păcurar D., Dinulescu A., Totu A.-V., Dijmărescu I., Pavelescu M.-L. (2025). *Escherichia coli* Urinary Tract Infections from a Romanian Pediatric Hospital: Antimicrobial Resistance Trends, ESBL Prevalence, and Empirical Treatment Implications. Antibiotics.

[51] Ajabnoor A.M., Thabit A.K., Alharbi O.S., Ibrahem K., Asiri T., Al-Ghamdi A.M., Felemban R.F. (2025). Epidemiology of Urinary Pathogens and Antibiotic Resistance Trends: A Retrospective Analysis Stratified by Age and Sex from a Large Tertiary Hospital in Saudi Arabia. Infect. Drug Resist..

[52] Guzmán-Blanco M., Labarca J.A., Villegas M.V., Gotuzzo E. (2014). Extended Spectrum β-Lactamase Producers among Nosocomial *Enterobacteriaceae* in Latin America. Braz. J. Infect. Dis..

[53] Ayzanoa B., Castillo-Vilcahuaman C., Salvatierra G., Dávila-Barclay A., Cuicapuza D., Huancachoque J., Calderón M., Rizo-Patrón E., Santillán-Salas C., Gilman R.H. (2026). Genomic Surveillance of Multidrug-Resistant *Escherichia coli* and *Klebsiella* in Clinical and Wastewater Isolates from a Paediatric Hospital in Lima, Peru. Access Microbiol..

[54] Caballero F., Martinez-Ventura A., Cuicapuza D., Fajardo-Loyola A., Gutierrez-Ajalcriña R., Soto-Pastrana J., Asmat-Marrufo P., de Vera E., Meza-Fernandez H., Chambi-Quispe M. (2025). Genomic Diversity of Uropathogenic *Escherichia coli* in Clinical Isolates from Six Latin American Countries, 2018–2023. Rev. Peru. Med. Exp. Salud Publica.

[55] Huang L., Hu H., Xu C., Zhou M., Li Y., Li Y., Wu S., Dong N. (2023). Characterization of NDM-5-Producing *Escherichia coli* Strains Isolated from Pediatric Patients with Bloodstream Infections in a Chinese Hospital. Genes.

[56] Linkevicius M., Bonnin R.A., Alm E., Svartström O., Apfalter P., Hartl R., Hasman H., Roer L., Räisänen K., Dortet L. (2023). Rapid Cross-Border Emergence of NDM-5-Producing *Escherichia coli* in the European Union/European Economic Area, 2012 to June 2022. Eurosurveillance.

[57] Juraschek K., Deneke C., Schmoger S., Grobbel M., Malorny B., Käsbohrer A., Schwarz S., Meemken D., Hammerl J.A. (2021). Phenotypic and Genotypic Properties of Fluoroquinolone-Resistant, Qnr-Carrying *Escherichia coli* Isolated from the German Food Chain in 2017. Microorganisms.

[58] Colom K., Pérez J., Alonso R., Fernández-Aranguiz A., Cisterna R. (2003). Simple and reliable multiplex PCR assay for detection of blaTEM, blaSHV and blaOXA-1 genes in Enterobacteriaceae. FEMS Microbiol. Lett..

