# Supplementary material for: Antimicrobial Resistance, β-Lactamases, and Virulence-Associated Genes in Clinical Escherichia coli Isolates from a Pediatric Tertiary-Care Hospital in Honduras
Source: Microorganisms. 2026 Oct 4;14(10):2257. doi: 10.3390/microorganisms14102257 (PMC13707494; doi:10.3390/microorganisms14102257)
Supplement: Supplementary file 1 [file microorganisms-14-02257-s001.zip › Figure S1 correlation matrices.pdf]

## Figure S1. Pairwise correlation matrices among antimicrobial resistance phenotypes and among virulence-associated genes.

### (A) Correlation matrix of non-susceptibility among the 11 antimicrobial agents evaluated.

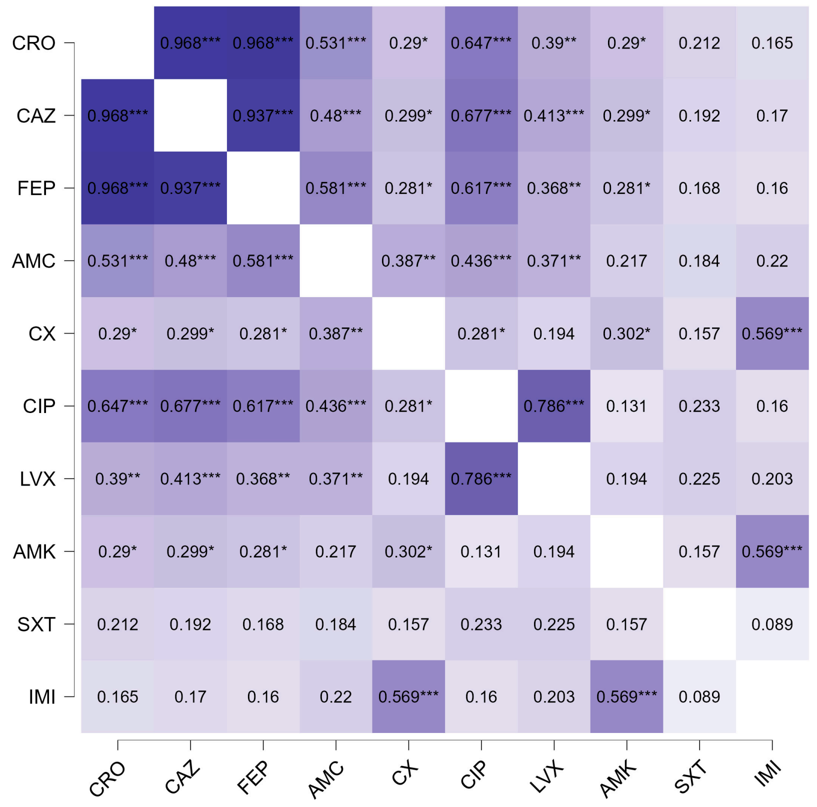


**Figure S1A.** Values shown are Pearson's correlation coefficients for binary variables (equivalent to the phi coefficient), calculated as described in Section 2.10 of the main text. Color intensity is proportional to the strength of the correlation. Asterisks indicate statistical significance (*, $p < 0.05$; **, $p < 0.01$; ***, $p < 0.001$). These correlations are indicative of co-occurrence patterns rather than definitive evidence of physical genetic linkage or a shared underlying resistance or virulence mechanism. Antibiotic abbreviations: CRO, ceftriaxone; CAZ, ceftazidime; FEP, cefepime; AMC, amoxicillin/clavulanic acid; CX, cefoxitin; CIP, ciprofloxacin; LVX, levofloxacin; AMK, amikacin; SXT, trimethoprim/sulfamethoxazole; IMI, imipenem. Virulence gene abbreviations are as defined in the main text.

## (B) Correlation matrix of the 17 virulence-associated genes evaluated.

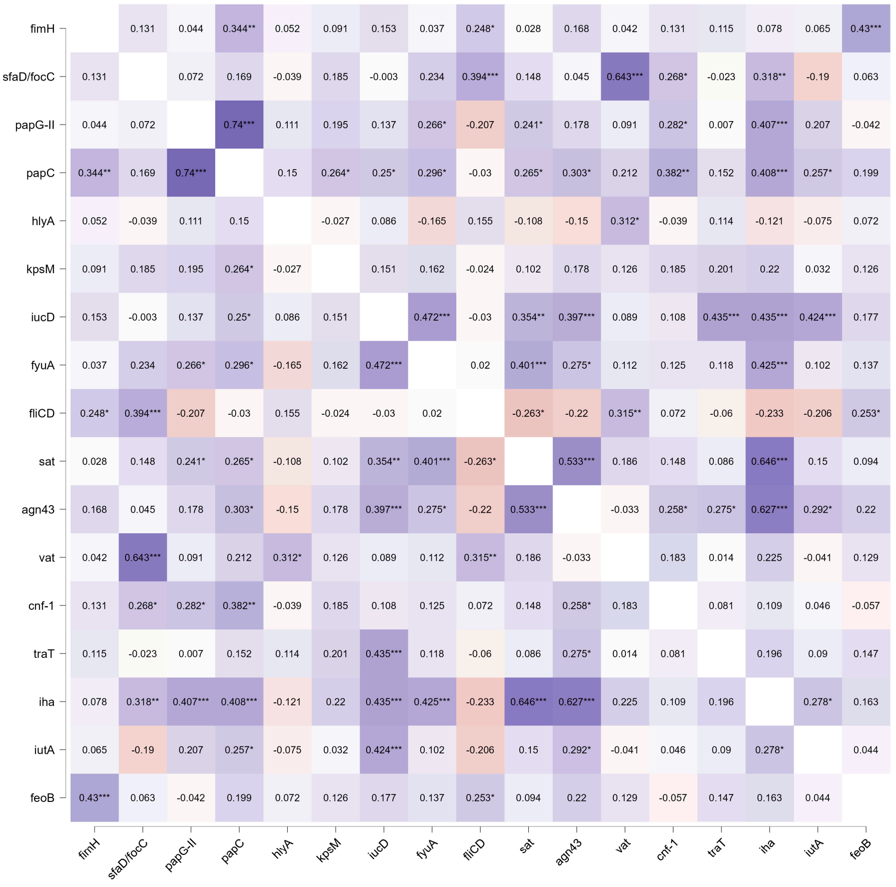


**Figure S1B.** Pairwise correlation matrix of the 17 virulence-associated genes evaluated in this study. Values shown are Pearson's correlation coefficients for binary variables (equivalent to the phi coefficient), calculated as described in Section 2.10 of the main text. Color intensity is proportional to the strength of the correlation. Asterisks indicate statistical significance (*, $p < 0.05$; **, $p < 0.01$; ***, $p < 0.001$). These correlations are indicative of co-occurrence patterns rather than definitive evidence of physical genetic linkage or a shared underlying virulence mechanism. Gene abbreviations: fimH, type 1 fimbrial adhesin; sfaD/focC, S/F1C fimbrial adhesin; papG-II, P fimbrial adhesin allele II; papC, P fimbrial assembly; hlyA, alpha-hemolysin; kpsM, group II capsule export; iucD, aerobactin biosynthesis; fyuA, yersiniabactin receptor; fliCD, flagellin-associated genes; sat, secreted autotransporter toxin; agn43, antigen 43; vat, vacuolating autotransporter toxin; cnf-1, cytotoxic necrotizing factor 1; traT, outer membrane protein associated with serum resistance; iha, iron-regulated gene A homolog adhesin; iutA, ferric aerobactin receptor; feoB, ferrous iron transport protein B.
