# Supplementary material for: Antimicrobial Resistance, β-Lactamases, and Virulence-Associated Genes in Clinical Escherichia coli Isolates from a Pediatric Tertiary-Care Hospital in Honduras
Source: Microorganisms. 2026 Oct 4;14(10):2257. doi: 10.3390/microorganisms14102257 (PMC13707494; doi:10.3390/microorganisms14102257)
Supplement: Supplementary file 1 [file microorganisms-14-02257-s001.zip › Supplementary Table S1.pdf]

Supplementary Table S1. Primers used in this study

Characteristic	Primer Name	Product	Sequence 5′ to 3′	Size product	Tm °C	Ref
Virulence	fimHF	*fimH*[a]	TTATGGCGGCGTGTTATC		54	1
	fimHR		TCCCTACTGCTCCTAACG	545 bp		
	sfaD/focCF	*sfaD/focC*[a]	AGGCAAATGGACAGGTATGG			
	sfaD/focCR		TCACCCAGAACAAACTTTCC	412 bp		
	papG-IIF	*papG-II*[a]	ATTCACCATAGAGGCGACTG			
	papG-IIR		ATCATTATGCGGCTCAGAC	237 bp		
	papCF	*papC*[a]	TTCTCTCTCCCTCAATACGG		60	
	papCR		TTATAACCTCAACGGGACGG	925 bp		
	fliCDF	*fliCD*[a]	CCGAATCAGAGTTAGTTCCG			
	fliCDR		CCCAGCGATGAAATACTTGC	610 bp		
	satF	*sat*[a]	GTTGGCAAACAGGTCAAAC			
	satR		CTCGGAGTATTGGCTTCAG	809 bp		
	hlyAF	*hlyA*[a]	GATACGCTGATAGGTGAG		58	
	hlyAR		CCAGGTGTGACTCAATAC	564 bp		
	kpsMF	*kpsM*[a]	CCAGAGTAGATATGACCAG			
	kpsMR		CTACGAGAAATACGAACAC	409 bp		
	agn43F	*agn43*[a]	CACACAGCCACTAATACC		58	
	agn43R		CACCTGAATACCCTTACC	488 bp		
	vatF	*vat*[a]	ATACAGTCTCGTCTCTGG			
	vatR		GTGACAGTCCCTTTATCC	670 bp		
	cnf-1F	*cnf-1*[c]	CAGACTCATCTTCACTCG			

	cnf-1R		AGACAGAGACCTTACGAC	551 bp		
	traTF	*traT* [c]	TGGTATAGTTCACATCTTCC			
	traTR		TAAAGCCTACTACTGGATTC	233 bp		
	fyuAF	*fyuA* [b]	CGCCAGTAAACAATCTTCCC			
	fyuAR		CCCAAACACCATATCAACGG	937 bp		
	iucDF	*iucD* [b]	CGTGAGACCCAGTTTATTTCC			
	iucDR		GGGCTGCTGAAGATATGAATAACC	334 bp		1
	iutAF	*iutA* [b]	GTTCACGCTCTTTGTCAGG			
	iutAR		GGGCTTAATCTCGGGAAAGG	801 bp	60	
	ihaF	*iha* [b]	TGTGCTCTGGTTTGATATGG			
	ihaR		CATTCTGGGTGCCTTATATCC	594 bp		
	feoBF	*feoB* [b]	GTCTAACCTTGAGCGTAACC			
	feoBR		GGCGAGGAAGATAGTCAGC	736 bp		
Identification	ybbWF	*ybbW*	TGATTGGCAAAATCTGGCCG			2,3
	ybbWR		ATACTGGCAATCAGTACGCC	667 bp		
MBL	NDM-F<br>NDM-R	bla_{NDM}	TGCATTGATGCTGAGCGGGTG<br>ATCACGATCATGCTGGCCTTG	685 bp	55	4
ESBL	SHV-F<br>SHV-R	bla_{SHV}	AGGATTGACTGCCTTTTTG<br>ATTTGCTGATTTCGCTCG	392 bp	50	5
	TEM-F<br>TEM-R	bla_{TEM}	CATTTCCGTGTCGCCCTTATTC<br>CGTTCATCCATAGTTGCCTGAC	799 bp	55	6
	CTX-M-F<br>CTX-M-R	bla_{CTX-M} group IV	GCTGGAGAAAAGCAGCGGAG<br>GTAAGCTGACGCAACGTCTG	474 bp	55	7

a: *E. coli* CFT073; **b:** *E. coli* ATCC 25922; **c:** UPEC 39

Reference

1. Ballesteros-Monrreal, M.G.; Arenas-Hernández, M.M.P.; Barrios-Villa, E.; Juarez, J.; Álvarez-Ainza, M.L.; Taboada, P.; De la Rosa-López, R.; Bolado-Martínez, E.; Valencia, D. Bacterial Morphotypes as Important Trait for Uropathogenic E. Coli Diagnostic; a Virulence-Phenotype-Phylogeny Study. *Microorganisms* **2021**, *9*, 2381, doi:10.3390/microorganisms9112381.
2. Carreón, E. Estudio Molecular de La Resistencia y Virulencia de Cepas de Escherichia Coli Productoras de β-Lactamasas de Espectro Extendido Aisladas de Vegetales Crudos. Master□s Thesis, Benemerita Universidad Autonoma de Puebla, 2019.
3. 37. Walker, D.I.; McQuillan, J.; Taiwo, M.; Parks, R.; Stenton, C.A.; Morgan, H.; Mowlem, M.C.; Lees, D.N. A Highly Specific Escherichia Coli QPCR and Its Comparison with Existing Methods for Environmental Waters. *Water Res.* **2017**, *126*, 101–110, doi:10.1016/j.watres.2017.08.032.
4. Hou, X., Song, X., Ma, X., Zhang, S., & Zhang, J. (2015). Molecular characterization of multidrug-resistant *Klebsiella pneumoniae* isolates. *Brazilian Journal of Microbiology*, 46(3), 759–768. https://doi.org/10.1590/S1517-838246320140138
5. Colom, K., Pérez, J., Alonso, R., Fernández-Aranguiz, A., & Cisterna, R. Simple and reliable multiplex PCR assay for detection of blaTEM, blaSHV and blaOXA-1 genes in Enterobacteriaceae. FEMS Microbiology Letters, 223(2), 147–151 (2003). https://doi.org/10.1016/S0378-1097(03)00306-9
6. Dallenne, C., Da Costa, A., Decré, D., Favier, C., & Arlet, G. (2010). Development of a set of multiplex PCR assays for the detection of genes encoding important β-lactamases in Enterobacteriaceae. *Journal of Antimicrobial Chemotherapy*, 65(3), 490–495. https://doi.org/10.1093/jac/dkp498
7. Pitout, J.D.D.; Hossain, A.; Hanson, N.D. Phenotypic and Molecular Detection of CTX-M-β-Lactamases Produced by Esche-richia Coli and Klebsiella Spp. J. Clin. Microbiol. 2004, 42, 5715–5721, doi:10.1128/JCM.42.12.5715-5721.2004.
